# Metabolic signatures of the integrated profile of cardiovascular autonomic modulation and cardiorespiratory fitness in apparently healthy individuals

**DOI:** 10.14814/phy2.70739

**Published:** 2026-01-26

**Authors:** Étore De Favari Signini, Alex Castro, Patrícia Rehder‐Santos, Juliana Cristina Milan‐Mattos, Juliana Magalhães de Oliveira, Alberto Porta, Renato Lajarim Carneiro, Antônio Gilberto Ferreira, Regina Vincenzi Oliveira, Aparecida Maria Catai

**Affiliations:** ^1^ Department of Physiotherapy Universidade Federal de São Carlos São Carlos São Paulo Brazil; ^2^ Department of Chemistry Universidade Federal de São Carlos São Carlos São Paulo Brazil; ^3^ Biosciences National Laboratory, Brazilian Center for Research in Energy and Materials São Carlos São Paulo Brazil; ^4^ Department of Biomedical Sciences for Health University of Milan Milan Italy; ^5^ Department of Cardiothoracic Vascular Anesthesia and Intensive Care, Policlinico San Donato Milan Italy

**Keywords:** autonomic nervous system, cardiorespiratory fitness, healthy volunteers, metabolomics

## Abstract

Cardiovascular autonomic modulation (CAM) and cardiorespiratory fitness (CRF) are well‐established predictors of health. Identifying metabolites associated with integrated CAM‐CRF profiles may help characterize healthy physiological states. This study aimed to investigate metabolic signatures representing distinct CAM‐CRF profiles in apparently healthy individuals. Non‐obese individuals (*n* = 127, 43 ± 14 years) underwent fasting blood collection for serum metabolome (SM) analysis, cardiovascular assessment, and a cardiopulmonary exercise test to access CAM and CRF. CAM‐CRF profiles were obtained separately by sex using principal components analysis (PCA) of CAM and CRF. Subjects' scores from the first two principal components of the PCA were used to generate the groups. Groups' SM were compared using one‐way ANOVA (controlling for age) and metabolite correlations were analyzed using the subjects' scores (controlling for age and body mass index), considering *p* < 0.01. In females, low sebacic acid levels were associated with high cardiac parasympathetic modulation (CPM) and greater cardiovascular complexity. In males, low ornithine levels corresponded to a profile with high CPM, baroreflex sensitivity (BRS), and CRF. Choline, betaine, N,N‐dimethylglycine levels in females, and glucose and sarcosine in males, were negatively correlated with CPM, BRS, CRF and cardiovascular complexity. These metabolites reflect integrated CAM‐CRF conditions, enhancing the understanding of underlying metabolic profiles.

## 1 INTRODUCTION

Cardiorespiratory fitness (CRF) and cardiovascular autonomic modulation (CAM) have been extensively studied in humans for over a century (Hill et al., 1924; Billman, 2011). This longstanding interest is based on the strong association of both CRF and CAM with overall health. These components provide valuable insights into the degree of impairment of organ systems, whether due to chronic degenerative diseases or aging process, as well as the body's ability to respond to physiological (e.g., physical activity, postural changes) and pathological (e.g., infectious diseases) stressors (Al‐Mallah et al., 2018; Fang et al., 2020; Fois et al., 2022; Goldenberg et al., 2019; Joyner & Casey, 2015; Porta et al., 2016; Raghuveer et al., 2020; Ross et al., 2016). Notably, both CRF and CAM are also linked to mortality risk (Imboden et al., 2018; Souza et al., 2021; Task Force, 1996), further reinforcing their relevance in biomedical research.

Alterations in CRF and CAM are frequently accompanied by metabolic changes (Kelly et al., 2020; Mathew et al., 2019; Signini, Castro, Rehder‐Santos, Cristina Millan‐Mattos, et al., 2022; Ziegler et al., 2021). The emergence of powerful disciplines such as metabolomics has enhanced our ability to explore these interactions in depth (Signini, Castro, Rehder‐Santos, Cristina Millan‐Mattos, et al., 2022; Signini, Castro, Rehder‐Santos, Milan‐Mattos, et al., 2022). Metabolomics enables the identification, quantification, and characterization of metabolites, which are small molecular intermediates (e.g., substrates or cofactors) and metabolic products. Metabolomics offer direct phenotypic information by profiling the metabolome, which encompasses the complete set of metabolites present in a biological samples such as bodily fluids or biological tissues (Griffiths et al., 2010; Nicholson & Lindon, 2008; Waller et al., 2020; Zhang et al., 2018). The blood serum metabolome (SM), in particular, reflects systemic physiological conditions, and consequently, holds promise for clarifying the complex interplay between CRF, CAM, and metabolic processes.

Although the relationships between SM, CAM, and CRF has been studied in different contexts (Kelly et al., 2020; Mathew et al., 2019; Signini, Castro, Rehder‐Santos, Cristina Millan‐Mattos, et al., 2022; Signini, Castro, Rehder‐Santos, Milan‐Mattos, et al., 2022; Ziegler et al., 2021), an integrate score combining CAM and CRF metrics (CAM‐CRF profile) in a weighted manner has not yet been evaluated. The CRF, defined as the capacity of the cardiorespiratory system to delivery oxygen to muscle tissue (Raghuveer et al., 2020), is commonly assessed via the highest oxygen consumption achieved during a maximal exercise test, called peak oxygen consumption (V̇O_2PEAK_). The CAM, on the other hand, reflects the sympathetic and parasympathetic modulation on the heart and vessels (for the latter, only sympathetic modulation) and is typically evaluated through measurements of resting cardiovascular oscillations of heart period (HP), systolic arterial pressure (SAP) values, and by estimating baroreflex sensitivity (BRS) (Catai et al., 2020; Milan‐Mattos et al., 2018; Porta et al., 2015). These cardiovascular oscillations, when assessed through symbolic analysis of HP and SAP variabilities, may also provide valuable information on the complexity of cardiovascular control (Porta et al., 2001; Wessel et al., 2000). Synthesizing these variables into composite score for each individual may offer a novel approach for identifying serum metabolic signatures that characterize CAM‐CRF profiles.

A more integrated understanding of physiological processes is crucial, as health outcomes arise from simultaneous and systemic responses. Furthermore, combining CRF and CAM – two health‐related components‐ into a single weighted score and associating this score with the serum metabolome may reveal metabolic signatures that reflect the organism's overall health. Therefore, finding serum metabolites that represent these profiles could help in future research aimed at simplifying access to the level of response to physical stress and systemic health (without the need for complex tests and analyses to identify a person's CAM‐CRF profile) through the development of specific blood tests to measure these metabolites. Thus, this study aimed to identify distinct CAM‐CRF profiles and investigate the metabolic signatures associated with these profiles in apparently healthy individuals.

## 2 METHODS

### 2.1 Participants

Participants were recruited through electronic and printed media, as well as from the database of the Cardiovascular Physiotherapy Laboratory (LFCV). Untrained individuals of both sexes were eligible if they were apparently healthy, had no diagnosed diseases, had a body mass index (BMI) <30 kg.m^−2^, were non‐smokers and non‐alcohol drinkers for at least 1 year, were not using medications or illicit drugs, and were between 20 and 70 years of age. Individuals who exhibited cardiovascular alterations during testing, including arterial hypertension, symptomatic arterial hypotension, severe and/or complex arrhythmias, or ST‐segment depression, were excluded. A total of 127 individuals (71 men and 56 women) were included in the study.

### 2.2 Ethical aspects

This study was approved by the Human Research Ethics Committee of the Federal University of São Carlos (UFSCar) (protocol number: 173/2011) and conducted in accordance with the standards set by the Declaration of Helsinki. All participants provided written informed consent prior to participation.

### 2.3 Experimental design

Following initial evaluation (anamnesis and physical examination) and informed consent, participants began the experimental protocol. Individuals whose last ergometric test had been conducted more than 1 year prior underwent a repeat test, supervised by a cardiologist at the School Health Unit of the Federal University of São Carlos (UFSCar) before continuing with the study assessments.

On the first day, each participant underwent morning blood collection and afternoon cardiovascular data acquisition for CAM analysis. On the second day, scheduled in close proximity to the first, participants completed a cardiopulmonary exercise test (CPET). Except for blood collection, all tests were performed at the LFCV. Blood collection for metabolomic and biochemical analysis was performed at a specialized clinical laboratory in São Carlos.

### 2.4 Blood samples

Blood was collected in the morning following a 12‐h fasting period. Participants were instructed to avoid strenuous physical activity for 48 h prior to blood collection. Blood samples were drawn using serum separator tubes (S‐Monovette 4.9 mL, Sarstedt, Germany). A portion of the sample was immediately transported to the university for serum extraction, while the remaining portion was processed at the specialized clinical laboratory for complete blood counts, lipid profiling, and other health‐related biochemical indices (Table 1).

**TABLE 1:** Characteristics of the participants.

Variables	All _(*n* = 127)_	Female _(*n* = 56)_	Male _(*n* = 71)_	*p*_(sex)_
Age _(years)_ ^a^	42 (30–55)	43 (31–56)	41 (30–51)	0.651
Height _(m)_	1.68 ± 0.09	1.61 ± 0.07	1.74 ± 0.07	**<0.001**
Weight _(kg)_	70.93 ± 12.11	62.74 ± 9.44	77.38 ± 9.91	**<0.001**
BMI _(kg/m2)_	24.98 ± 2.85	24.16 ± 2.94	25.62 ± 2.62	**0.005**
Erythrocytes _(million/mm3)_ ^a^	4.90 (4.60–5.10)	4.51 (4.40–4.80)	5.10 (4.97–5.30)	**<0.001**
Hemoglobin _(g/dl)_	14.53 ± 1.19	13.59 ± 0.88	15.27 ± 0.82	**<0.001**
Hematocrit _(%)_ ^a^	42.40 (40.40–44.30)	40.10 (38.30–41.40)	44.00 (42.45–45.35)	**<0.001**
Leukocytes _(mm3)_	5955 ± 1387	5865 ± 1466	6026 ± 1329	0.395
Lymphocytes _(mm3)_	2042 ± 560	2009 ± 458	2068 ± 631	0.847
Monocytes _(mm3)_ ^a^	464 (404–569)	444 (403–516)	482 (410–611)	0.105
TC _(mg/dl)_	197.56 ± 44.05	200.50 ± 50.61	195.24 ± 38.31	0.617
HDL _(mg/dl)_	58.00 ± 21.36	68.34 ± 25.67	49.85 ± 12.27	**<0.001**
LDL _(mg/dl)_ ^a^	114.00 (93.00–132.00)	108.00 (86.75–123.00)	121.00 (100.00–139.50)	0.010
VLDL _(mg/dl)_ ^a^	21.00 (14.00–29.00)	16.00 (13.00–25.00)	23.00 (17.00–30.00)	**0.009**
Triglycerides _(mg/dl)_ ^a^	97.00 (69.00–139.50)	77.00 (63.00–112.50)	117.00 (84.00–149.50)	**<0.001**
Uric Acid _(mg/dl)_	5.30 ± 1.42	4.15 ± 0.99	6.20 ± 0.99	**<0.001**
Creatinine _(mg/dl)_	0.90 ± 0.18	0.74 ± 0.09	1.02 ± 0.13	**<0.001**
Glucose _(mg/dl)_	92.27 ± 8.48	89.77 ± 7.77	94.24 ± 8.55	**0.001**
Blood urea _(mg/dl)_	32.81 ± 7.73	30.23 ± 7.90	34.85 ± 6.99	**<0.001**
hs‐CRP _(mg/L)_	1.20 ± 1.97	1.69 ± 2.72	0.81 ± 0.92	0.144

*Note*: Data are mean ± standard deviation. Independent *t*‐test with *p* < 0.01. Bold *p*‐values indicate statistical significance.

Abbreviations: BMI, body mass index; HDL, high density lipoprotein; hs‐CRP, high‐sensitivity C‐reactive protein; LDL, low density lipoprotein; TC, total cholesterol; VLDL, very low density lipoprotein.

^a^ Mann–Whitney test (data presented as median and interquartile range).

At the university, blood samples were centrifuged at 1450*g* for 10 min (Sorvall ST 8 Benchtop Centrifuge, Thermo Scientific, Massachusetts, USA) (Signini, Castro, Rehder‐Santos, Cristina Millan‐Mattos, et al., 2022). The resulting serum was aliquoted and stored at −80°C until analysis.

### 2.5 Cardiovascular autonomic assessment

Cardiovascular data collection was always performed in the afternoon. Participants were instructed to continue abstaining from physical exercise as previously requested for blood sampling. Additionally, they were advised to avoid stimulant and alcoholic foods and beverages (e.g., coffee, stimulant teas, energy drinks, alcoholic beverages, and high‐sugar foods) for 24 h prior to assessment. On the day of testing, participants were advised to refrain from consuming meals with excessive protein and fat, and to ensure adequate sleep the night before. They were also instructed not to arrive fasting (i.e., >5 h since the last meal) or immediately after eating (<2 h post‐meal). Women of reproductive age were evaluated between the 7th and 10th days of their menstrual cycle. Only individuals with regular cycles or who had been postmenopausal for at least 1 year were included. Each participant rested supine for 10 min prior to data collection. Then, cardiovascular and respiratory data, including heart rate, blood pressure, and respiratory rate were continuously recorded for 10 min. During this time, participants were instructed to remain still, silent, and awake. Ambient temperature was maintained between 21°C and 24°C, with relative humidity kept between 40% and 60% (Catai et al., 2020).

Electrocardiogram (ECG) signals were acquired via MC5 lead (BioAmp FE132, ADInstruments, New South Wales, Australia). Beat‐to‐beat arterial pressure was measured using non‐invasive finger photophethysmography (Finometer Pro, Finapres Medical Systems, Netherlands). Respiratory movements were recorded through a thoracic belt (Marazza, Monza, Italy). Analog‐to‐digital conversion was carried out by the acquisition device Power Laboratory 8/35 hardware (ADInstruments, New South Wales, Australia) and analyzed with LabChart software, version 7.3.8 (ADInstruments, New South Wales, Australia).

### 2.6 Cardiopulmonary exercise testing (CPET)

CPET was conducted as described by Signini et al. (Signini, Castro, Rehder‐Santos, Cristina Millan‐Mattos, et al., 2022) and De Maria et al.(de Maria et al., 2019). Briefly, the CPET was performed using an incremental protocol on a treadmill ergometer (Master ATL, Inbramed, Rio Grande do Sul, Brazil). The test was performed either until the participant reached exhaustion or an interruption criterion outlined by Balady et al., (2010). The highest V̇O_2_ value recorded during the final 30 s of the CPET was considered as V̇O_2PEAK_ (de Maria et al., 2019). Ventilatory and metabolic variables were obtained breath‐by‐breath using a metabolic cart (ULTIMA MedGraphics—St Paul, Minesota, USA) and processed via Breeze Suite 7.1 software (MedGraphics—St. Paul, Minesota, USA). Concurrently, 12‐lead ECG readings were collected using an electrocardiograph (CardioPerfect, Welch Allyn, New York, USA).

### 2.7 Autonomic data processing

Stable sequences (the most stable part of the total data collection) of 256 consecutive HP and SAP values were selected according to the principle of short‐term variability analysis (Catai et al., 2020; Lombardi et al., 2018; Pagani et al., 1986; Task Force, 1996). The HP and SAP fluctuations provides information about autonomic modulation directed to the heart and vessels (Catai et al., 2020; Lombardi et al., 2018; Pagani et al., 1986; Task Force, 1996; Porta et al., 2015). Series were carefully checked to avoid erroneous detections or missed beats. Isolated ectopic beats were corrected using linear interpolation between values unaffected by the arrhythmic beat (Catai et al., 2020; de Maria et al., 2019).

HP and SAP variabilities were analyzed in the frequency domain. This analysis computed the spectral power of series according to the autoregressive model (Porta et al., 1998). The spectral components were classified as very low‐frequency (VLF, below 0.04 Hz), low‐frequency (LF, from 0.04 to 0.15 Hz), and high‐frequency (HF, from 0.15 to 0.40 Hz) (Task Force, 1996). The power of each component of HP series was expressed in absolute units (ms^2^) and in normalized units, namely the relative value in percentage of HF and LF powers in proportion to the total spectral power minus the VLF power (HF_nu_ and LF_nu_). HF and HF_nu_ represent cardiac parasympathetic modulation (CPM), while LF and LF_nu_ powers were taken as an indication of cardiac sympathetic modulation (CSM) (Pagani et al., 1996; Task Force, 1996). Finally, the ratio between the LF and HF (LF/HF) was used to typify the sympatho‐vagal balance over the heart. Spectral analysis was carried out over the SAP series as well. Only LF and LF_nu_ powers were computed on SAP series and they were taken as markers of sympathetic modulation on the vessels (Pagani et al., 1986).

Univariate symbolic analysis (Guzzetti et al., 2005; Porta et al., 2001) was also used to evaluate CAM. Briefly, stable sequences with HP and SAP values were uniformly quantized over six levels (0–5). Each original value was substituted with a symbol coding the quantization level it belongs to. Then, patterns composed by three consecutive symbols were built. Patterns were classified into four families and were created to classify HP and SAP variations according to variations between symbols: no variation (0 V), namely all symbols are the same (e.g., 4 4 4 or 2 2 2); one variation (1 V), namely two consecutive symbols are the same, and the remaining symbol differs (e.g., 3 4 4 or 4 4 2); two like variations (2LV) with the three consecutive symbols forming an ascending or descending ramp (e.g., 1 2 4 or 4 3 2); two unlike variations (2UV) with the three consecutive symbols forming a peak or valley (e.g., 2 4 2 or 4 1 2). The method computed the occurrence rates of these patterns, expressed as 0 V_%_, 1 V_%_, 2LV_%_, and 2UV_%_. These percentages were obtained by dividing the number of patterns in each family by the total number of patterns (multiplied by 100). The 2LV_%_ and 2UV_%_ represent CPM when considering the HP series, while the 0 V_%_ represents CSM to the sinus node and vessels when computed over the HP and SAP series respectively (Porta et al., 2015). The 1 V_%_ provides information on mixed modulation (sympathetic and parasympathetic) of HP (Porta et al., 2001).

Complexity of the HP and SAP variability series was computed as the Shannon entropy (SE) of the distribution of the patterns formed by three consecutive symbols (Porta et al., 2001). The SE is an index that describes the shape of the pattern distribution and provides a quantification of the complexity of the pattern distribution (Porta et al., 2001). When the distribution is flat, the SE is large as all the patterns are equally present and complexity of the series is high. When there is a subset of patterns that are most likely, while others are absent or uncommon, the SE is small (Porta et al., 2001) as well as complexity. High SE values indicate a more adaptable and flexible cardiovascular control (Valencia et al., 2009).

The baroreflex sensitivity (BRS) was calculated using the sequence method (de Maria et al., 2019; Porta et al., 2000). The baroreflex origin pattern was defined as a joint HP‐SAP scheme with three consecutive and contemporaneous increases or decreases in both HP and SAP. The HP and SAP ramps with zero‐beat latency were paired and considered of baroreflex origin. We followed the recommendation given in (Porta et al., 2013) that suggests to retain all detected HP‐SAP patterns of baroreflex origin, regardless of the magnitude of total or partial SAP and HP variations and the strength of the linear association between HP and SAP values. We checked that the results did not differ from those obtained after following the prerequisites given in Bertinieri et al. (1985). The BRS was calculated as the mean slope of the HP‐SAP regression lines over all patterns of baroreflex origin (*α*_seq_). BRS was positive and expressed in ms·mmHg^−1^. We also evaluated the percentage of HP‐SAP patterns of baroreflex origin in relation to the total number of HP‐SAP patterns (SEQ_%_) taken as an indication of the degree of cardiac baroreflex control involvement (with SEQ_%_ ranging from 0 to 100).

### 2.8 Metabolomics analyses

Blood serum samples were processed and analyzed using hydrogen nuclear magnetic resonance (^1^H NMR) and liquid chromatography coupled to high‐resolution mass spectrometry (LC‐HRMS) to obtain small molecules (SM) metabolomic profiles, following protocols described by Castro et al. (2022) and Signini, Castro, Rehder‐Santos, Cristina Millan‐Mattos, et al. (2022) (Castro et al., 2022; Signini, Castro, Rehder‐Santos, Cristina Millan‐Mattos, et al., 2022).

For ^1^H NMR, sample filtrates were obtained by using 3 kDa filter tubes (Amicon Ultra) and centrifuging at 14,000*g* for 30 min at 4°C. A total volume of 100 μL of filtered serum was transferred to a 5 mm NMR tube (Wilmad Standard Series 5 mm, Sigma‐Aldrich) containing 40 μL of phosphate buffer (monobasic sodium phosphate, NaH2PO4, 119.97 g·mol^−1^; dibasic sodium phosphate, Na2HPO4, 141.96 g·mol^−1^, 0.1 M, pH 7.4), TMSP‐d4 (3‐(trimethylsilyl)‐2,2′,3,3′‐tetradeuteropropionic acid), and 260 μL of D2O (99.9%; Sigma‐Aldrich, San Luis, CA, USA). The concentration of the internal reference (TMSP‐d4) in the NMR tube was 0.5 mmol·L^−1^. Spectra were acquired using a 600 MHz Bruker spectrometer (14.1 Tesla) equipped with a 5 mm TCI cryoprobe at 298 K. The pulse sequence with H_2_O presaturation signal (named noesypr1d by Bruker) was used adopting a continuous wave for the 1H‐spectrum. Acquisition parameters included: spectral width (SW = 30 ppm), acquisition time (AQ = 3.63 s), 90° pulse duration (p1 = 9.5 μs), relaxation delay (d1 = 4 s), and number of scans (ns = 128). Baseline corrections, metabolite characterization, and quantification were performed using Chenomx Suite 8.6 software (Chenomx Inc., Edmonton, AB, Canada).

For the LC‐HRMS, 150 μL of serum were mixed with 450 μL of cold methanol in a new tube for metabolite extraction. After storage at −20°C for 5 min, samples were vortexed for 20 seconds and centrifuged at 7267*g* at 4°C for 10 min. A 200 μL aliquot of the supernatant was transferred to a new tube, and 20 μL of the internal standard solution (5 mM L‐leucine‐enkephalin acetate) was added. Samples were stored at −20°C until transferred to vials for LC‐HRMS analysis. Blank samples (samples composed of solvents and processed identically to experimental samples) and quality control (QC) samples (a pool mix of 15 μL from each experimental sample) were also prepared. An Agilent UHPLC system (model 1290 Infinity II) consisting of a binary LC‐pump (G7120A), an autosampler (G7129B), and temperature‐controlled column compartment (G7116) was used. Mass analysis was performed using a high‐resolution QqTOF instrument (Impact HD QTOF™, Bruker Daltonics) with electrospray ionization (ESI) operated in positive and negative modes. Software used for MS and MS/MS data acquisition and analysis included HyStar, Compass QTOF Control 3.4, Data Analysis 4.2, and Profile Analysis 2.1 (Bruker Daltonics). Liquid chromatography separation employed an Eclipse XDB‐C18 Agilent column (100 × 3.0 mm i.d; 3.5 μm). The featured bucket was generated using Bruker Profile Analysis 2.1 software with the following parameters: S/N threshold = 2; minimum acquisition of 10 spectra; correlation coefficient threshold = 0.2; and smoothing width = 1. Inclusion criteria required: values >5% of the blank sample values; coefficient of variation (CV) <20% for QC samples; and <10% missing data in experimental samples and <20% in QC samples. Local non‐linear regression (Loess) normalization (Tsugawa et al., 2014, 2015) was applied to remaining features to assess instrumental stability (Figure S1) before subsequent analysis. Data Analysis 4.2 (Bruker Daltonics) was used to conduct MS/MS ion fragment identification. Putative identifications of fragment ions were determined by comparing statistically significant fragments ions against HMDB MS/MS database (https://hmdb.ca, version 5.0), Mass Bank (https://massbank.eu/MassBank/, version 2.2.4), Mass Bank of North America (https://mona.fiehnlab.ucdavis.edu/, version 3.0), and CEU Mass Mediator (http://ceumass.eps.uspceu.es/, version 3.0).

### 2.9 CAM‐CRF score generation

Principal component analysis (PCA) was applied to V̇O_2PEAK_ and CAM variables (LF, HF, LF_nu_, HF_nu_, LF/HF, 0V_%_, 1V_%_, 2LV_%_, 2UV_%_, α_seq_ and SEQ_%_) derived from both HP and SAP series. Unlike the short‐term HP sequences, only 0V_%_, LF and LF_nu_ from the short‐term SAP sequences were included in the PCA, as they are the only indices related to sympathetic autonomic modulation on the vessels. The SE from the short‐term HP and SAP sequences were also included in the PCA.

The PCA is a multivariate statistical method of dimensionality reduction that transforms highly dimensional and complex datasets into less dimensional and complex ones (Fávero & Belfiore, 2017; Jolliffe & Cadima, 2016). It is important to note that PCA does not require distributional assumptions, making it an adaptive exploratory method (Jolliffe & Cadima, 2016). In PCA, the dimensional reduction process occurs by creating new variables, called principal components (PCs), while preserving as much variability as possible (Fávero & Belfiore, 2017). The PCs are orthogonal (which means they are complementary) to each other and exhibit linear functions that are consistent with those observed in the initial dataset (Jolliffe & Cadima, 2016).

The number of PCs is equal to the number of original variables included in the PCA. Each PC captures (or explain) a percentage of the total data variability (Fávero & Belfiore, 2017). However, they capture different percentages and are ranked accordingly. The percentage captured is inversely proportional to the PC number and therefore, PC_1_ always assesses the largest portion of the total variability, PC_2_ always the second largest, and so on until the last PC generated (Fávero & Belfiore, 2017). Consequently, the first PCs describe the main influencing factors of the sample studied, while the last PCs describe “noise” or residual variabilities.

**FIGURE 1:**
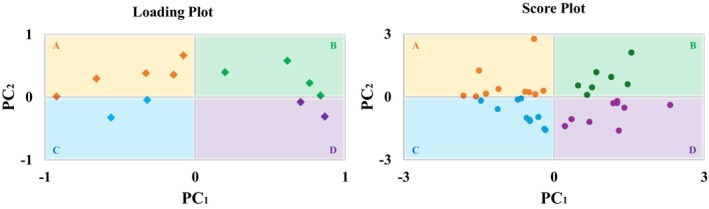
Loading Plot: This plot highlights the variables included in the PCA (diamonds) and their respective importance. The variables that are farther away from the center are more important in explaining the variance of the data in the respective PCs. Score Plot: Shows the scores generated for each individual analyzed in the PCA (circles) based on the values of all the variables and their importance in the analyzed PCs. Note that variables represented in quadrant A (loading plot) have higher values in individuals located in quadrant A (score plot), variables represented in quadrant B (loading plot) have higher values in individuals located in quadrant B (score plot), and so on (Fávero & Belfiore, 2017). The quadrants of the score plot were considered groups, and each individual's scores were used in subsequent analyses. PC, principal component; PCA, principal component analysis.

The PCA generates scores (for each observation) and loadings (for each original variable), which are typically represented in score and loading plots (Figure 1) (Fávero & Belfiore, 2017; Jolliffe & Cadima, 2016). Firstly, the score is the position of a given observation within a given PC (Figure 1) (Jolliffe & Cadima, 2016). This position is defined according to a linear combination of all included variables, weighted by the loadings, for a given PC. In this sense, the score plot shows the position of each observation based on the PCs included in the plot (e.g., PC_1_ and PC_2_) (Figure 1) (Fávero & Belfiore, 2017). Secondly, the computed loadings indicate how much each original variable contributes to the data variability for a given PC (Fávero & Belfiore, 2017; Jolliffe & Cadima, 2016). The loadings are computed as the Pearson correlation between the original variable values and the given PC (Fávero & Belfiore, 2017). In this sense, the stronger the correlation between an original variable and a PC, the more important that variable is in explaining the variation in the data for that PC (Fávero & Belfiore, 2017). Thus, the loading plot shows the importance of each original variable based on the PCs included in the plot (e.g., PC_1_ and PC_2_) (Figure 1). Additionally, it is noteworthy that “communalities” represent a sum of loadings of a given set of PCs (e.g., PC_1_ + PC_2_ + …), which makes it possible to determine the importance of a given variable in explaining a larger proportion of total variability (Fávero & Belfiore, 2017).

It is important to note that loadings range in value from −1 to 1. Briefly, a negative loading value on a given PC indicates an inverse relationship between a given original variable and the PC. A positive loading value indicates a direct relationship between a given original variable and a given PC (Fávero & Belfiore, 2017). Considering this information, along with the fact that scores represent the location of observations within a given PC, scores with positive values within a given PC are associated with higher values of the original variables with positive loadings values on that PC, and lower values of the original variables with negative loadings values on the same PC (Fávero & Belfiore, 2017; Jolliffe & Cadima, 2016). These characteristics underscore the importance of evaluating the loading and scoring plots together when performing a PCA.

In light of the above explanation, the variables represented in the loading plot in the present study were derived from CAM and CRF, as previously described. The observations in the score plot were the study participants. We chose to use only PC_1_ and PC_2_ because they provide the most relevant information about the variability of the data. We determined the quadrants based on the central values of each PC (value “0”) with PC_1_ on the x‐axis and PC_2_ on the y‐axis. Thus, the quadrants were generated and represent the integration of CAM and CRF measures and they reflect distinct CAM‐CRF profiles. The quadrants of the score plot were then treated as groups for comparative analysis of their SMs profiles (Figure 1).

### 2.10 Statistical analysis

Normality and homoscedasticity were tested using Shapiro–Wilk and Levene's tests, respectively, across all variables (SM metabolites, V̇O_2PEAK_, CAM indices, CAM‐CRF scores, and participant characteristics). If assumptions were violated, the Box‐Cox transformation were employed.

Given the pronounced influence of sex on the analyses, PCA was performed separately for males and females. Commonality was extracted from the first two PCs, and the contribution of each CAM and CRF variable to total variance was examined. SM profiles, CAM variables, and V̇O_2PEAK_ of the four groups (formed by CAM‐CRF scores within each sex) were compared using one‐way analysis of variance (Holm‐Sidak's post hoc test), adjusting for age as a covariate. If normality and homoscedasticity for the application of the Holm–Sidak test were not passed despite the application of Box‐Cox transformation, the Kruskal–Wallis test with Bonferroni's correction was conducted. In addition, differences between sexes were assessed via independent *t*‐test or Mann–Whitney test, as appropriate. To explore associations between CAM‐CRF scores and serum metabolites, partial correlation analyses were performed after adjusting for age and BMI. Partial correlation analyses were carried out separately on male and female groups as well.

All statistical tests applied a significance threshold of *p* < 0.01 to account for multiple comparisons (Signini, Castro, Rehder‐Santos, Cristina Millan‐Mattos, et al., 2022). Analyses were performed using SPSS software, version 25.0.

## 3 RESULTS

### 3.1 Participant characteristics and biochemical profile

No significant differences in age were observed between males and females participants (Table 1). Except for HDL cholesterol, all significant variables related to blood tests and anthropometric measures presented lower values in females compared to males (Table 1). Overall, the values obtained from blood analysis were within normal or borderline reference ranges, even when the individuals were separated into the analyzed groups (Tables S1 and S2) (American Diabetes Association, 2022; Faludi et al., 2017; Pearson et al., 2003; Rosenfeld et al., 2019; Sui et al., 2008).

All other variables used for the comparison between sexes, including CAM indices, CRF parameters, and metabolite profiles, are detailed in Table S3.

### 3.2 CAM‐CRF score

**FIGURE 2:**
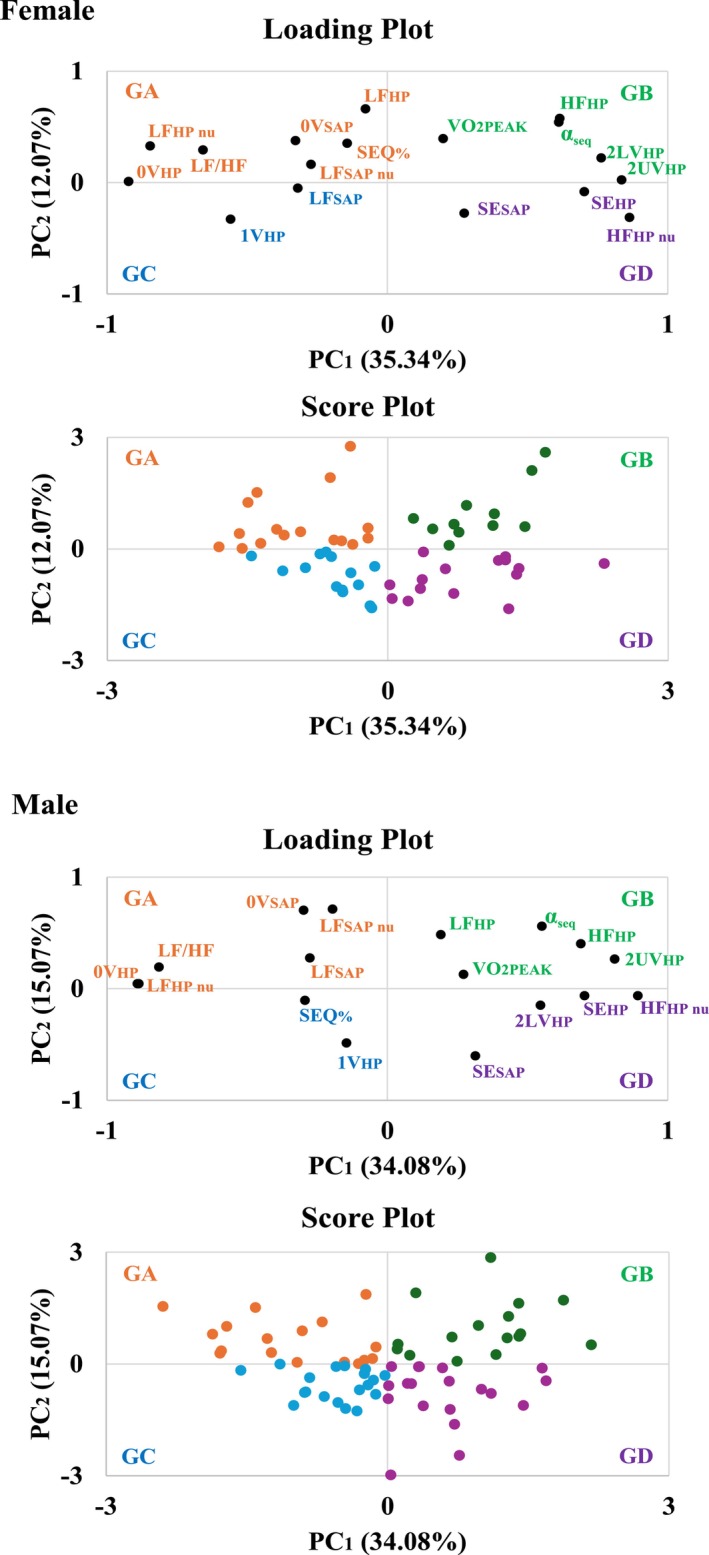
Loading and score plots generated by PCA for both sexes. Loading plot: The farther a variable is from the center (value 0), the more important it was to the variability of the corresponding PCs. Score plot: The farther the score is from the center (value 0), the more important the variables in the same quadrant (in the loading plot) were in determining the score value. 0V, No variation; 1V, One variation; 2LV, Two like variations; 2UV, Two unlike variations; α_seq_, Baroreflex sensitivity (BRS); GA, Group A; GB, Group B; GC, Group C; GD, Group D; HF, high frequency band; HF_nu_, HF in normalized unites; HP, heart period; LF, low frequency band; LF_nu_, LF in normalized unites; PC, principal component; PCA, principal component analysis; SAP, systolic arterial pressure; SE, Shannon entropy; SEQ, percentage of HP‐SAP patterns of baroreflex origin; V̇O_2PEAK_, peak oxygen consumption.

The first two PCs (PC_1_ and PC_2_) accounted for over 45% of the total sample variability, 47.41% in females and 49.15% in males (Figure 2). Communality analysis (Table 2) indicated that 0V_HP_, 2UV_HP_, LF_nu HP_ and HF_nu HP_ were the primary contributors to the variability captured by these components across both sexes. Notably, baroreflex sensitivity (BRS) emerged as a key contributor in females, while the LF/HF was particularly relevant in males (Table 2).

**TABLE 2:** Communalities considering the first two PCs in the female and male group.

Variable	Extraction _(Female)_	Extraction _(Male)_
SE_HP_	0.500	0.498
0V_HP (%)_	0.852	0.788
1V_HP (%)_	0.420	0.259
2LV_HP (%)_	0.631	0.321
2UV_HP (%)_	0.698	0.728
LF_HP (ms2)_	0.445	0.272
LF_HP (nu)_	0.824	0.796
HF_HP (ms2)_	0.669	0.639
HF_HP (nu)_	0.843	0.802
LF/HF_HP_	0.518	0.701
SE_SAP_	0.151	0.463
0V_SAP (%)_	0.250	0.584
LF_SAP (ms2)_	0.104	0.152
LF_SAP (nu)_	0.101	0.546
SEQ _(%)_	0.146	0.097
α_seq (ms•mmHg−1)_	0.711	0.618
V̇O_2PEAK (ml/kg/min)_	0.196	0.090

*Note*: Values range from 0 to 1. The closer the variable is to the value “1”, the greater its importance in explaining the variability of the data observed in the sum of the selected PCs (considering, in this case, only the sum of the variances explained in PC_1_ and PC_2_).

Abbreviations: 0V, no variation; 1V, one variation; 2LV, two like variations; 2UV, two unlike variations; α_seq_, baroreflex sensitivity (BRS); HF, high frequency band; HFnu, HF in normalized unites; HP, heart period; LF, low frequency band; LFnu, LF in normalized unites; SAP, systolic arterial pressure; SE, Shannon entropy; SEQ, percentage of HP‐SAP patterns of baroreflex origin; V̇O_2PEAK_, peak oxygen consumption.

Based on PC scores, participants were stratified into four groups: Group A (GA), Group B (GB), Group C (GC), and Group D (GD), for both sexes (Figure 2). The CAM‐CRF characteristics of each generated group were similar between the sexes (Table 3). GA was associated with CSM; GB, characterized by elevated CRF, CPM, and BRS; GC, displayed mixed autonomic modulation; and GD, linked to high complexity of cardiovascular control and CPM.

**TABLE 3:** Characterization of groups generated by PCA.

Group	PC_1 (loading signal)_	PC_2 (loading signal)_	Typical variables _(female)_	Typical variables _(male)_	Characteristics
GA	Negative	Positive	0V_HP_/0V_SAP_/LF_HP_/LF_nu HP_/LF/HF/LF_nu SAP_/SEQ_%_	0V_HP_/0V_SAP_/LF_nu HP_/LF/HF/LF_SAP_/LF_nu SAP_	Predominantly CSM
GB	Positive	Positive	V̇O_2PEAK_/α_seq_/HF_HP_/2LV_HP_/2UV_HP_	V̇O_2PEAK_/α_seq_/LF_HP_/HF_HP_/2UV_HP_	Predominantly CPM and high BRS and CRF
GC	Negative	Negative	1V_HP_/LF_SAP_	1V_HP_/SEQ_%_	Mixed autonomic modulation
GD	Positive	Negative	SE_SAP_/SE_HP_/HF_nu HP_	2LV_HP_/SE_SAP_/SE_HP_/HF_nu HP_	High cardiovascular complexity and CPM

Abbreviations: 0V, no variation; 1V, one variation; 2LV, two like variations; 2UV, two unlike variations; α_seq_, baroreflex sensitivity (BRS); CPM, cardiac parasympathetic modulation; CRF, cardiorespiratory fitness; CSM, cardiovascular sympathetic modulation; GA, group A; GB, group B; GC, group C; GD, group D; HF, high frequency band; HF_nu_, HF in normalized unites; HP, heart period; LF, low frequency band; LF_nu_, LF in normalized unites; PC, principal component; PCA, principal component analysis; SAP, systolic arterial pressure; SE, Shannon entropy; SEQ, percentage of HP‐SAP patterns of baroreflex origin; V̇O_2PEAK_, peak oxygen consumption.

BMI did not differ significantly across groups (females: *p* = 0.155; males: *p* = 0.711) (Tables S1 and S2). However, age showed significant group difference in males (*p* = 0.005), with lower values observed in GB compared to GA (*p* = 0.009) and GC (*p* = 0.009) (Table S2). Age differences in females approached significance (*p* = 0.012) (Table S1). All other characterization variables showed no significant differences between generated groups for both sexes. The CAM and V̇O_2PEAK_ index values included in the PCA for each group (male and female) are presented in Tables 4 and 5. As expected, due to the nature of the PCA, the groups differed significantly from each other in terms of the CAM‐CRF profile.

**TABLE 4:** Indices and variables used in the generation of CAM‐CRF scores, including their respective values across the defined groups for females.

Variables	Female				
	GA _(*n* = 16)_	GB _(*n* = 11)_	GC _(*n* = 14)_	GD _(*n* = 15)_	*p*
SE_HP_	3.38 ± 0.29^**BD**^	3.88 ± 0.48^**A**^	3.39 ± 0.27^**D**^	3.97 ± 0.25^**AC**^	**<0.001**
0V_HP (%)_ ^a^	27.17 (22.24–32.78)^**BD**^	6.69 (3.94–10.63)^**AC**^	24.41 (20.37–26.67)^**BD**^	4.33 (2.36–11.61)^**AC**^	**<0.001**
1V_HP (%)_	46.87 ± 4.52	41.12 ± 6.32^**C**^	50.17 ± 3.45^**BD**^	43.70 ± 6.13^**C**^	**<0.001**
2LV_HP (%)_	10.68 ± 5.63^**BD**^	20.44 ± 6.68^**AC**^	9.53 ± 3.43^**BD**^	19.21 ± 5.46^**AC**^	**<0.001**
2UV_HP (%)_	14.12 ± 5.78^**BD**^	30.64 ± 10.42^**AC**^	15.58 ± 5.27^**BD**^	29.82 ± 8.83^**AC**^	**<0.001**
LF_HP (ms2)_	1399.45 ± 1741.02^**C**^	1056.13 ± 794.81	205.38 ± 140.78^**A**^	357.65 ± 238.34	**0.002**
LF_HP (nu)_ ^a^	80.87 (66.23–87.64)^**BD**^	36.22 (29.90–50.18)^**A**^	52.53 (45.21–70.21)	37.68 (21.87–46.42)^**A**^	**<0.001**
HF_HP (ms2)_	398.16 ± 503.43^**B**^	1811.88 ± 1403.43^**AC**^	151.11 ± 127.80^**BD**^	682.94 ± 348.75^**C**^	**<0.001**
HF_HP (nu)_	22.54 ± 14.45^**BCD**^	58.76 ± 12.62^**A**^	41.41 ± 13.41^**AD**^	63.89 ± 16.12^**AC**^	**<0.001**
LF/HF_HP_	5.68 ± 4.77^**BD**^	0.72 ± 0.46^**A**^	1.69 ± 1.12^**AD**^	0.63 ± 0.38^**AC**^	**<0.001**
SE_SAP_	3.32 ± 0.28^**D**^	3.29 ± 0.30^**D**^	3.37 ± 0.24	3.64 ± 0.27^**AB**^	**0.002**
0V_SAP (%)_	36.34 ± 10.01^**D**^	33.36 ± 10.47^**D**^	28.49 ± 7.45	21.13 ± 9.52^**AB**^	**<0.001**
LF_SAP (ms2)_	8.97 ± 9.34	2.79 ± 1.82	10.14 ± 15.86	3.33 ± 3.51	0.238
LF_SAP (nu)_	71.41 ± 19.59	62.48 ± 14.63	69.83 ± 23.08	58.58 ± 20.15	0.323
SEQ_(%)_	4.79 ± 4.09	3.21 ± 2.86	2.81 ± 1.98	2.00 ± 1.43	0.027
α_seq (ms•mmHg‐1)_	13.00 ± 5.78^**B**^	27.49 ± 9.25^**ACD**^	9.63 ± 4.27^**B**^	15.44 ± 9.07^**B**^	**<0.001**
V̇O_2PEAK (ml/kg/min)_	26.56 ± 6.29	31.12 ± 6.04	24.80 ± 6.16	24.93 ± 3.40	0.131

*Note*: Data are mean ± standard deviation. One‐way ANOVA with Sidak's post hoc test controlled for age and *p* < 0.01. Bold *p*‐values indicate statistical significance.

Abbreviations: 0V, no variation; 1V, one variation; 2LV, two like variations; 2UV, two unlike variations; αseq, baroreflex sensitivity (BRS); ^**A**^, significant differences with GA; ^**B**^, significant differences with GB; ^**C**^, significant differences with GC; ^**D**^, significant differences with GD; GA, group A; GB, group B; GC, group C; GD, group D; HF, high frequency band; HFnu, HF in normalized unites; HP, heart period; LF, low frequency band; LFnu, LF in normalized unites; SAP, systolic arterial pressure; SE, Shannon entropy; SEQ, percentage of HP‐SAP patterns of baroreflex origin; V̇O_2PEAK_, peak oxygen consumption.

^a^ Kruskal–Wallis test (data presented as median and interquartile range).

**TABLE 5:** Indices and variables used in the generation of CAM‐CRF scores, including their respective values across the defined groups for males.

Variables	Male				
	GA _(*n* = 17)_	GB _(*n* = 16)_	GC _(*n* = 19)_	GD _(*n* = 19)_	*p*
SE_HP_	3.23 ± 0.40^**BD**^	3.77 ± 0.20^**AC**^	3.32 ± 0.29^**BD**^	3.74 ± 0.32^**AC**^	**<0.001**
0V_HP (%)_	35.11 ± 12.52^**BD**^	9.97 ± 6.49^**AC**^	29.53 ± 7.35^**BD**^	14.84 ± 6.70^**AC**^	**<0.001**
1V_HP (%)_	44.53 ± 6.50	42.25 ± 7.08^**C**^	49.21 ± 3.79^**B**^	48.2 ± 4.35	**0.002**
2LV_HP (%)_	9.31 ± 5.26	13.36 ± 5.46	9.12 ± 3.21	13.74 ± 5.47	0.035
2UV_HP (%)_ ^a^	10.24 (8.66–13.78)^**BD**^	33.46 (24.70–43.80)^**AC**^	11.81 (10.04–13.58)^**BD**^	20.08 (16.54–29.92)^**AC**^	**<0.001**
LF_HP (ms2)_	815.56 ± 642.35	980.47 ± 683.50	518.68 ± 479.68	525.95 ± 580.82	0.016
LF_HP (nu)_	77.88 ± 14.17^**BD**^	40.32 ± 16.50^**AC**^	68.63 ± 11.74^**BD**^	42.76 ± 18.83^**AC**^	**<0.001**
HF_HP (ms2)_	280.37 ± 360.46^**B**^	1634.18 ± 1227.38^**AC**^	248.80 ± 274.74^**B**^	735.49 ± 855.45	**<0.001**
HF_HP (nu)_	21.32 ± 14.73^**BD**^	58.41 ± 16.11^**AC**^	30.49 ± 11.99^**BD**^	56.22 ± 18.74^**AC**^	**<0.001**
LF/HF_HP_	6.04 ± 4.25^**BD**^	0.83 ± 0.60^**AC**^	3.04 ± 2.33^**BD**^	1.01 ± 0.85^**AC**^	**<0.001**
SE_SAP_	3.15 ± 0.32^**D**^	3.26 ± 0.26^**D**^	3.41 ± 0.28	3.58 ± 0.22^**AB**^	**<0.001**
0V_SAP (%)_	43.24 ± 10.68^**CD**^	39.47 ± 11.23^**D**^	30.65 ± 8.71^**A**^	24.16 ± 11.48^**AB**^	**<0.001**
LF_SAP (ms2)_	18.85 ± 23.21	7.32 ± 5.39	8.55 ± 11.43	5.22 ± 7.49	0.024
LF_SAP (nu)_ ^a^	90.93 (86.69–94.49)^**CD**^	88.24 (79.39–90.42)^**D**^	64.32 (58.09–81.31)	67.41 (46.92–76.76)^**AB**^	**<0.001**
SEQ_(%)_	5.35 ± 3.47^**B**^	2.56 ± 1.72^**AC**^	5.00 ± 3.25^**B**^	3.65 ± 2.57	**0.002**
α_seq (ms•mmHg‐1)_	13.81 ± 8.52	26.09 ± 12.48^**C**^	10.56 ± 5.27^**B**^	13.91 ± 5.95	**0.004**
V̇O_2PEAK (ml/kg/min)_	31.79 ± 5.71	39.76 ± 6.68	33.42 ± 6.07	36.42 ± 6.43	0.050

*Note*: Data are mean ± standard deviation. One‐way ANOVA with Sidak's post hoc test controlled for age and *p* < 0.01. Bold *p*‐values indicate statistical significance.

Abbreviations: 0V, no variation; 1V, one variation; 2LV, two like variations; 2UV, two unlike variations; αseq, baroreflex sensitivity (BRS); ^**A**^, significant differences with GA; ^**B**^, significant differences with GB; ^**C**^, significant differences with GC; ^**D**^, significant differences with GD; GA, group A; GB, group B; GC, group C; GD, group D; HF, high frequency band; HFnu, HF in normalized unites; HP, heart period; LF, low frequency band; LFnu, LF in normalized unites; SAP, systolic arterial pressure; SE, Shannon entropy; SEQ, percentage of HP‐SAP patterns of baroreflex origin; V̇O_2PEAK_, peak oxygen consumption.

^a^ Kruskal–Wallis test (data presented as median and interquartile range).

The metabolomic profile of each sex and of each group generated according to the CAM‐CRF profile for both sexes was evaluated. Forty‐seven metabolites were identified and quantified by ^1^H NMR (Table S3 and Figure S2), alongside 37 additional molecules detected by LC‐HRMS following methodological filters (Tables S3–S6). Among these compounds, 21 were identified and found to be significant between the sexes (Table 6). All these metabolites had higher values in male individuals, except for creatine and glycine.

**TABLE 6:** Identified metabolites with significantly different serum levels between the sexes.

Metabolites	Female _(*n* = 56)_	Male _(*n* = 71)_	*p*
^*1*^ *H NMR*_(mM)_			
2‐Hydroxyisovalerate	0.007 ± 0.003	0.011 ± 0.004	**<0.001**
3‐Hydroxyisobutyrate	0.015 ± 0.004	0.018 ± 0.007	**0.002**
Betaine^a^	0.045 (0.038–0.050)	0.052 (0.044–0.059)	**0.001**
Choline	0.010 ± 0.002	0.012 ± 0.003	**0.005**
Creatine^a^	0.037 (0.025–0.045)	0.017 (0.011–0.027)	**<0.001**
Creatinine	0.071 ± 0.013	0.099 ± 0.015	**<0.001**
Glycine^a^	0.343 (0.293–0.428)	0.313 (0.256–0.359)	**0.004**
Histidine	0.082 ± 0.017	0.090 ± 0.015	**0.006**
Isoleucine	0.065 ± 0.011	0.078 ± 0.018	**<0.001**
Lactate	2.852 ± 0.865	3.331 ± 0.805	**0.001**
Leucine	0.089 ± 0.018	0.107 ± 0.019	**<0.001**
Lysine	0.140 ± 0.023	0.151 ± 0.023	**0.006**
Methionine	0.028 ± 0.005	0.031 ± 0.005	**<0.001**
N,N‐Dimethylglycine^a^	0.003 (0.002–0.003)	0.004 (0.003–0.004)	**<0.001**
Phenylalanine	0.069 ± 0.008	0.076 ± 0.010	**<0.001**
Proline^a^	0.207 (0.176–0.245)	0.248 (0.222–0.296)	**<0.001**
Trimethylamine^a^	0.001 (0.001–0.002)	0.002 (0.002–0.003)	**<0.001**
Valine	0.236 ± 0.043	0.267 ± 0.050	**<0.001**
*LC‐HRMS*_(a.u.)_			
D‐Phenylalanine^a^	0.866 (0.804–0.972)	0.963 (0.885–1.063)	**0.002**
Hydroxyphenyllactic acid	0.786 ± 0.349	1.204 ± 0.596	**<0.001**
L‐Tryptophan^a^	0.889 (0.802–0.954)	1.002 (0.888–1.095)	**<0.001**

*Note*: Data are mean ± standard deviation. Independent *t*‐test with *p* < 0.01. Bold *p*‐values indicate statistical significance.

Abbreviations: ^1^H NMR, hydrogen nuclear magnetic resonance; LC‐HRMS, liquid chromatography coupled to high‐resolution mass spectrometry.

^a^ Mann–Whitney test (data presented as median and interquartile range).

**FIGURE 3:**
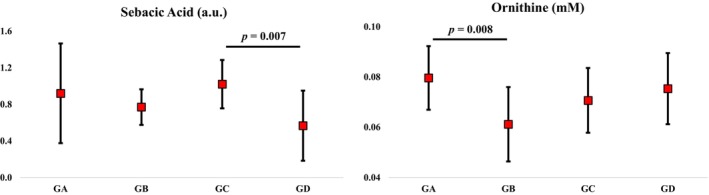
Serum sebacic acid and ornithine in female and male individuals, respectively. Data presented as mean and standard deviation. GA, Group A; GB, Group B; GC, Group C; GD, Group D. One‐way ANOVA with Sidak's post hoc test controlled for age and *p* < 0.01.

When considering the groups generated by PCA for each sex (Tables S4 and S5), after adjusting for age covariate, only sebacic acid (females, *p* = 0.008) and ornithine (males, *p* = 0.008), along with an unknown molecule detected via LC‐HRMS (males), showed significant group differences (Figure 3). GC displayed higher sebacic acid levels than GD in females (*p* = 0.007), while GA showed elevated ornithine compared to GB in males (*p* = 0.008).

**FIGURE 4:**
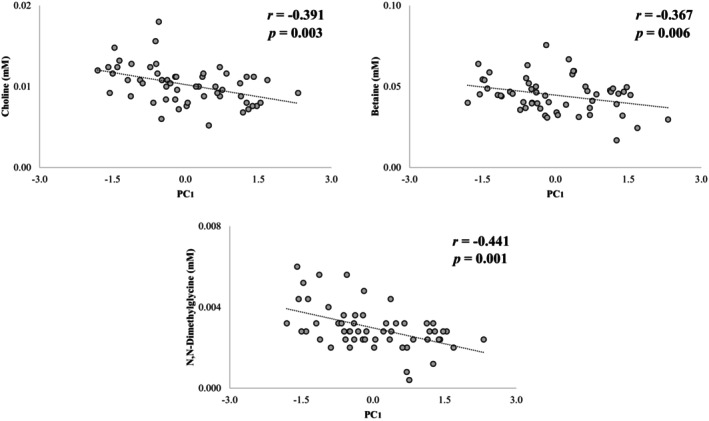
Significant partial correlations (controlled by the covariates age and BMI) were observed between serum levels of choline, betaine, and N,N‐dimethylglycine, with the scores obtained in PC_1_, for females. Negative PC_1_ values indicate a predominance of CSM, while positive values indicate a predominance of CPM, as well as greater complexity of cardiovascular control, BRS, and CRF (see Table 3). BMI, body mass index; BRS, baroreflex sensitivity; CPM, cardiac parasympathetic modulation; CRF, cardiorespiratory fitness; CSM, cardiovascular sympathetic modulation; PC, principal component. *p* < 0.01.

**FIGURE 5:**
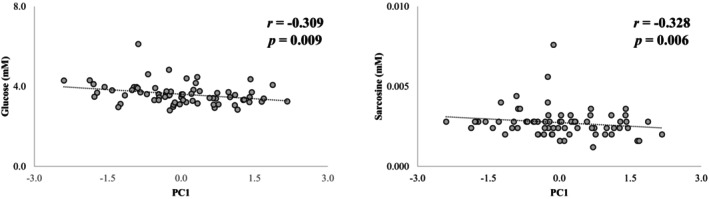
Significant partial correlations (controlled by the covariates age and BMI) were observed between serum levels of glucose and sarcosine, with the scores obtained in PC_1_, for males. Negative PC_1_ values indicate a predominance of CSM, while positive values indicate a predominance of CPM, as well as greater complexity of cardiovascular control, BRS, and CRF (see Table 3). BMI, body mass index; BRS, baroreflex sensitivity; CPM, cardiac parasympathetic modulation; CRF, cardiorespiratory fitness; CSM, cardiovascular sympathetic modulation; PC, principal component. *p* < 0.01.

Correlations between PC scores and metabolic variables were evaluated with age and BMI as covariates. In females, PC_1_ was negatively associated with betaine, choline, and *N,N*‐dimethylglycine (*p* = 0.006, 0.003, and 0.001, respectively; Figure 4). In males, PC_1_ was negatively associated with glucose and sarcosine (*p* = 0.009 and 0.006, respectively; Figure 5). No significant associations were found between PC_2_ and any known metabolites.

## 4 DISCUSSION

This study aimed to determine different integrated profiles of CAM and CRF, and to identify metabolic signatures associated with these profiles in apparently healthy individuals. In the present study, we identified significant associations between CAM‐CRF profiles and seven metabolites: sebacic acid, ornithine, choline, betaine, *N,N*‐dimethylglycine, sarcosine, and glucose. Among these, sebacic acid and ornithine emerged as particularly relevant, as they characterize distinct CAM‐CRF profiles and contributed more substantially to overall data variability.

Sebacic acid, a medium‐chain fatty acid (MCFA), can be endogenously synthesized or obtained through dietary sources (Liao et al., 2024). This metabolite serves as an energy substrate via β‐oxidation and exhibits glycemic regulatory and anti‐inflammatory properties (Liao et al., 2024; Ogawa et al., 2024). Recently, sebacic acid has been proposed as a potential biomarker of aging due to its reduced serum levels with advancing age (Huang et al., 2023). However, in our study, lower sebacic acid levels were observed in females with greater complexity of cardiovascular control and elevated CPM (i.e., superior cardiovascular health). This finding seems contradictory given that good glycemic control and anti‐inflammatory effects are positively associated with cardiovascular health. However, a recent study comparing fasting serum levels of sebacic acid across three groups of individuals (non‐obese, obese, and diabetic) reported a positive correlation between fasting levels of this metabolite and blood glucose during the oral glucose tolerance test (OGTT) (Tripolt et al., 2023). These data suggest that elevated sebacic acid may be associated with a worse OGTT, and, consequently, to a metabolic limitation in glucose metabolism (Tripolt et al., 2023). Notably, the beneficial effects of sebacic acid were primarily observed through diet supplementation or food‐based intake rich in this metabolite (Liao et al., 2024). Therefore, we speculate that endogenous sebacic acid may be a compensatory response to impaired glucose metabolism. Taken together, we believe that reduced serum sebacic acid levels in individuals with greater complexity of cardiovascular control may reflect enhanced metabolic flexibility, which is closely associated with metabolic and cardiovascular health.

Ornithine emerged as a critical metabolite distinguishing male groups with high CSM (GA), versus those exhibiting elevated CPM, CRF, and BRS (GB). Ornithine plays a key role in the urea cycle and is associated with tissue fibrosis, cancer, and inflammation (Sivashanmugam et al., 2017). This is primarily due to ornithine's relationship with arginase activity, an enzyme that catalyzes the conversion of arginine to ornithine and mediates inflammatory processes. Moreover, ornithine serves as a precursor for proline, an important metabolite in collagen production, and polyamines, metabolites involved in cell proliferation (Karadima et al., 2025; Sivashanmugam et al., 2017; Szondi et al., 2021). It is important to note that arginase activity increases in immune cells during inflammatory stimuli (e.g., pro‐inflammatory interleukins), and elevated levels may be indicative of chronic inflammatory states (Karadima et al., 2025; Li et al., 2021). Although the effects of increased serum ornithine levels in the absence of diagnosed diseases are still unclear, a study showed that low arginine‐to‐ornithine ratio has been associated with a significantly increased cardiovascular mortality (Ishinoda et al., 2023). These facts support our findings that individuals with lower serum ornithine levels may reflect a high CPM, CRF, and BRS in males.

PC_1_ scores showed inverse relationships with serum levels of choline, betaine, and *N,N*‐dimethylglycine in females, as well as with glucose and sarcosine levels in males. Choline and betaine have beneficial effects on human physiology. For example, choline contributes to the synthesis of the neurotransmitter acetylcholine, which regulates autonomic modulation (primarily within the parasympathetic autonomic modulation branch) (Liu Chung Ming et al., 2024). Betaine controls oxidative stress and inflammation by regulating homocysteine levels (Arumugam et al., 2021; Zhao et al., 2018), while *N,N*‐dimethylglycine has also been shown to enhance immune response and improve tissue oxygen consumption (Gray & Titlow, 1982; Kendall, 1994). Although the negative relationships observed with PC_1_ scores may appear contradictory (given that negative PC_1_ values are associated with CSM, whereas positive values are linked to greater complexity of cardiovascular control, CPM, BRS, and CRF), it is important to note that the physiological benefits of these metabolites are typically observed in the context of supplementation or physical activity interventions (Arumugam et al., 2021; Pathmasiri et al., 2024; Ueland, 2011). Under natural conditions, elevated serum levels of these metabolites may be associated with impaired utilization/transportation capacity of these compounds across tissues, and may be associated with cardiovascular, metabolic, and neurological dysfunctions (Bye et al., 2012; Marino et al., 2025; Svingen et al., 2015). Interestingly, these metabolites, together with sarcosine, which showed marginal significance in females, *p* = 0.019, comprise the mitochondrial arm of the choline degradation pathway, suggesting that mitochondrial disturbances may underlie unfavorable CAM‐CRF profiles.

Only male individuals showed a negative correlation between metabolites glucose and sarcosine and PC_1_. Blood glucose levels as a health biomarker are well‐established (Joseph et al., 2022). Individuals with diabetes and insulin resistance tend to have lower resting CAM and CRF values than healthy individuals with similar lifestyle habits (de Moura‐Tonello et al., 2016; Röhling et al., 2017; Hamaoka et al., 2023; Macedo et al., 2023). However, it is noteworthy that the individuals whose demonstrated worse glucose levels were free of any diagnosed disease. This underscores the importance of blood glucose levels, even in healthy individuals. Finally, elevated sarcosine levels have been associated with prostate cancer (Koutros et al., 2013; Walters et al., 2018). However, it remains unclear whether elevated sarcosine is a cause or a consequence of these metabolic contexts, since it may also be related to longevity and proteostatic adaptations (Walters et al., 2018). Its dual origins, from the *N,N‐*dimethylglycine catabolism and glycine methylation via glycine *N‐*methyltransferase, may partly explain the differences in outcomes between the sexes in our cohort (Ueland, 2011).

Sex influenced the serum metabolome of the participants. As shown in Table 6, the differences mainly occurred in the levels of amino acids and their derivatives. Except for creatine and glycine, all other metabolites had higher serum levels in males. This finding is consistent with previous research and can be primarily explained by the fact that male individuals naturally have greater muscle mass, a higher creatine‐to‐phosphocreatine conversion, and a higher nutrient intake than female (Bordoni et al., 2020; Costanzo et al., 2022; Rist et al., 2017). Notably, some of these metabolites were associated with the CAM‐CRF profile in females individuals, further emphasizing the physiological differences between the sexes.

The significant metabolites observed in the main analyses of this study suggest a link to specific CAM‐CRF profiles and offers insights into overall health. The CAM and CRF are closely related to the body's ability to respond to physiological and pathological stressors (Al‐Mallah et al., 2018; Fois et al., 2022; Joyner & Casey, 2015; Porta et al., 2016; Raghuveer et al., 2020). It is important to emphasize that measurements of both CAM and CRF depend on the effective integration of multiple systems, including the nervous, musculoskeletal, metabolic, cardiovascular, and respiratory systems (Hansen et al., 1999; Porta et al., 2015). Not surprisingly, both are also associated with mortality risk, with higher resting CSM and lower CRF and complexity of cardiovascular control predicting a worse prognosis (Fang et al., 2020; Imboden et al., 2018; Souza et al., 2021; Task Force, 1996). Thus, the analysis of CAM and CRF provides a comprehensive overview of an individual's systemic health. In this context, sebacic acid and ornithine merit attention because they characterize groups with distinct CAM‐CRF profiles based on two PCs, accounting for at least 47.41% of total data variability. Lower sebacic acid values highlight high complexity of cardiovascular control and high CPM in females, while lower ornithine values highlight high CRF, BRS, and CPM, and low CSM in males. Metabolites of the choline degradation pathway are also important for females, indicating that higher serum values are associated with a worse health profile (higher resting CSM and lower CRF, CPM, BRS and complexity of cardiovascular control). In males, serum glucose and sarcosine exhibit a similar pattern to the metabolites from the choline degradation pathway. Therefore, we emphasize that these metabolites have the potential be related with systemic health and deserve further investigation.

This study had some limitations. The sample size was relatively small, and a combined analysis of both sexes was not feasible. Additionally, the primary results were based on only a portion of the total data variability (based only on PCs 1 and 2), and only a limited number of metabolites were evaluated. Nevertheless, this study employed a robust analytical methodology that differed from conventional approaches. Furthermore, the results were based on comprehensive study whereas metabolites were obtained via two distinct analytical techniques. This study did not account for the food records of the subjects included or their physical activity level. However, all subjects were placed under the same conditions on the day of blood collection: 12 h of fasting, restriction of certain foods and drinks, and restriction of strenuous physical activity.

## 5 CONCLUSION

In non‐obese apparently healthy individuals, elevated fasting serum levels of sebacic acid, ornithine, choline, betaine, *N,N*‐dimethylglycine, sarcosine, and glucose are associated with poorer global health, marked by higher CSM and lower CRF, lower CPM, and lower complexity of cardiovascular control. These metabolites reflect integrated CAM‐CRF conditions, enhancing the understanding of underlying metabolic profiles in apparently healthy individuals and may represent promising metabolic indicators of systemic health. They deserve further investigation to assess their clinical prognostic value.

## FUNDING INFORMATION

This study was supported by the São Paulo Research Foundation (FAPESP) (#2023/09582‐4, #2023/01626‐2, #2016/22215‐7, and #2014/50244‐6), the National Council for Scientific and Technological Development (CNPq) (#151218/2023‐4) and the Coordination for the Improvement of Higher Education (CAPES grant number: 001). The funding agencies had no participation in the study design, data collection and analysis, or manuscript preparation.

## CONFLICT OF INTEREST STATEMENT

The authors have no competing interest to declare.

## Supporting information


Data S1.

## Data Availability

The omics datasets generated and/or analyzed during the current study are available at the following link: https://doi.org/10.6084/m9.figshare.30866009.
