# Supplementary material for: Metabolic signatures of the integrated profile of cardiovascular autonomic modulation and cardiorespiratory fitness in apparently healthy individuals
Source: Physiol Rep. 2026 Jan 26;14(2):e70739. doi: 10.14814/phy2.70739 (PMC12835618; doi:10.14814/phy2.70739)
Supplement: Supplementary file 1 — Data S1. [file PHY2-14-e70739-s001.pdf]

Metabolic signatures of the integrated profile of cardiovascular autonomic modulation and cardiorespiratory fitness in apparently healthy individuals

Étore De Favari Signini[1]; Alex Castro[2,3]; Patrícia Rehder-Santos[1]; Juliana Cristina Milan-Mattos[1]; Juliana Magalhães de Oliveira[2]; Alberto Porta[4,5]; Renato Lajarim Carneiro[2]; Antônio Gilberto Ferreira[2]; Regina Vincenzi Oliveira[2]; Aparecida Maria Catai[1,*].

[1] Department of Physiotherapy, Universidade Federal de São Carlos, São Carlos, São Paulo, Brazil.

[2] Department of Chemistry, Universidade Federal de São Carlos, São Carlos, São Paulo, Brazil.

[3] Biosciences National Laboratory, Brazilian Center for Research in Energy and Materials, Campinas, São Paulo, Brazil.

[4] Department of Biomedical Sciences for Health, University of Milan, Milan, Italy.

[5] Department of Cardiothoracic, Vascular Anesthesia and Intensive Care, Policlinico San Donato, San Donato Milanese, Milan, Italy.

Keywords: Metabolomics, Autonomic Nervous System, Cardiorespiratory Fitness, Healthy Volunteers.

***Corresponding author**: Aparecida Maria Catai, PhD, Department of Physical Therapy, Cardiovascular Physical Therapy Laboratory, Nucleus of Research in Physical Exercise, Federal University of São Carlos, Via Washington Luiz, km 235, CP: 676, 13565-905, São Carlos-SP, Brazil, Tel: +55 16 33518705, Fax: +55 16 33612081, email: mcatai@ufscar.br. Co-corresponding author: signinietof@gmail.com (Étore De F. Signini)

Competing Interests: The authors have no competing interest to declare.

SUPPORTING INFORMATION

Supplementary Table 1. Characteristics of female participants in each generated group.

Supplementary Table 2. Characteristics of male participants in each generated group.

Supplementary Table 3. Sex-based differences in CAM, CRF and metabolic indices/variables.

Supplementary Table 4. Metabolic variables detected and measured by ^{1}H NMR and LC-HRMS analytical techniques for each group evaluated for female individuals.

Supplementary Table 5. Metabolic variables detected and measured by ^{1}H NMR and LC-HRMS analytical techniques for each group evaluated for male individuals.

Supplementary Table 6. LC-HRMS data for statistically significant features identified in serum samples.

Supplementary Figure 1. Assessment of Instrumental stability using quality control samples.

Supplementary Figure 2. Baseline adjustment performed with *Chenomx Suite 8.6* software and quantification of significant metabolites via ^{1}H NMR spectroscopy.

Supplementary Table 1. Characteristics of female participants in each generated group.

Variables	GA (n = 16)	GB (n = 11)	GC (n = 14)	GD (n = 15)	*p*
Age (years)	45 ± 16	35 ± 11	53 ± 13	40 ± 14	0.012
BMI (kg/m2)	25.29 ± 1.93	22.69 ± 2.92	24.27 ± 3.77	23.94 ± 2.74	0.155
Erythrocytes (million/mm3)	4.68 ± 0.27	4.41 ± 0.25	4.56 ± 0.29	4.55 ± 0.34	0.152
Hemoglobin (g/dl)	13.91 ± 0.86	13.17 ± 0.77	13.71 ± 0.91	13.45 ± 0.88	0.164
Hematocrit (%)	40.83 ± 2.20	39.01 ± 1.39	40.34 ± 1.96	39.89 ± 2.35	0.242
Leukocytes (mm3)	5738 ± 1378	6038 ± 1541	5851 ± 1972	5885 ± 1027	0.943
Lymphocytes (mm3)	1987 ± 447	1961 ± 408	2084 ± 610	1997 ± 373	0.717
Monocytes (mm3)	423 ± 86	505 ± 209	482 ± 169	508 ± 101	0.206
TC (mg/dl)	209.31 ± 48.47	194.73 ± 68.62	211.93 ± 46.53	184.67 ± 40.73	0.661
HDL (mg/dl)	67.06 ± 34.04	74.73 ± 29.08	64.36 ± 13.18	68.73 ± 23.07	0.564
LDL (mg/dl)*	111.00 (105.25 - 129.75)	93.00 (67.00 – 102.00)	126.50 (101.75 - 139.25)	102.00 (61.00 - 111.50)	0.011
VLDL (mg/dl)*	18.00 (13.75 - 25.25)	15.00 (13.00 - 15.50)	22.00 (14.50 - 27.50)	15.00 (12.00 - 24.50)	0.552
Triglycerides (mg/dl)	91.06 ± 49.39	75.27 ± 45.99	113.21 ± 55.29	82.2 ± 38.88	0.740
Uric Acid (mg/dl)	4.38 ± 1.10	3.83 ± 0.85	4.36 ± 1.06	3.93 ± 0.87	0.521
Creatinine (mg/dl)	0.76 ± 0.07	0.75 ± 0.13	0.73 ± 0.10	0.74 ± 0.07	0.756
Glucose (mg/dl)	89.44 ± 10.46	86.09 ± 3.42	92.21 ± 8.09	90.53 ± 5.77	0.522
Blood urea (mg/dl)	29.44 ± 6.27	29.09 ± 6.25	34.29 ± 10.44	28.13 ± 7.16	0.661
hs-CRP (mg/L)	1.13 ± 1.09	1.31 ± 3.03	1.97 ± 2.75	2.30 ± 3.65	0.437

* Kruskal-Wallis test (data presented as median and interquartile range). Data are mean ± standard deviation. BMI: body mass index; HDL: high density lipoprotein; hs-CRP: high-sensitivity C-reactive protein; LDL: low density lipoprotein; TC: total cholesterol; VLDL: very low density lipoprotein. One-way ANOVA with Sidak's post hoc test with $p < 0.01$.

Supplementary Table 2. Characteristics of male participants in each generated group.

Variables	GA (n = 17)	GB (n = 16)	GC (n = 19)	GD (n = 19)	*p*
Age (years)	46 ± 13[B]	32 ± 8[AC]	46 ± 13[B]	43 ± 14	**0.005**
BMI (kg/m2)	26.19 ± 3.28	25.40 ± 1.63	25.42 ± 2.59	25.49 ± 2.80	0.711
Erythrocytes (million/mm3)	5.07 ± 0.29	5.10 ± 0.23	5.23 ± 0.63	5.15 ± 0.27	0.745
Hemoglobin (g/dl)	15.50 ± 0.69	15.03 ± 0.68	15.29 ± 1.02	15.26 ± 0.80	0.234
Hematocrit (%)	44.59 ± 2.50	43.53 ± 2.15	44.41 ± 2.43	44.73 ± 2.57	1.000
Leukocytes (mm3)	5726 ± 1175	6115 ± 1390	6189 ± 1124	6055 ± 1626	0.393
Lymphocytes (mm3)	1965 ± 451	2297 ± 781	1877 ± 503	2158 ± 711	0.781
Monocytes (mm3)	538 ± 118	459 ± 159	554 ± 205	490 ± 163	0.053
TC (mg/dl)	204.76 ± 34.67	189.75 ± 35.53	188.63 ± 45.60	197.95 ± 36.63	0.279
HDL (mg/dl)	49.18 ± 12.93	48.13 ± 7.48	50.05 ± 11.82	51.68 ± 15.62	0.898
LDL (mg/dl)	131.41 ± 27.96	117.50 ± 30.08	113.11 ± 37.11	117.53 ± 31.28	0.144
VLDL (mg/dl)	23.29 ± 11.28	23.38 ± 9.46	24.63 ± 13.64	28.11 ± 11.20	0.278
Triglycerides (mg/dl)	116.59 ± 56.10	117.00 ± 47.41	123.53 ± 67.92	140.74 ± 56.13	0.287
Uric Acid (mg/dl)	6.58 ± 1.09	5.93 ± 0.97	6.24 ± 0.87	6.06 ± 1.00	0.496
Creatinine (mg/dl)	1.05 ± 0.14	1.04 ± 0.13	0.99 ± 0.12	1.02 ± 0.12	0.521
Glucose (mg/dl)*	95.00 (87.00 – 100.00)	94.50 (87.50 – 98.00)	94.00 (91.00 – 97.00)	94.00 (91.00 - 96.50)	0.971
Blood urea (mg/dl)	35.94 ± 5.68	33.63 ± 6.20	35.53 ± 7.82	34.21 ± 8.04	0.838
hs-CRP (mg/L)	0.68 ± 0.82	0.43 ± 0.45	0.95 ± 1.14	1.12 ± 0.98	0.079

* Kruskal-Wallis test (data presented as median and interquartile range). Data are mean ± standard deviation. [A]: significant differences with GA; [B]: significant differences with GB; BMI: body mass index; [C]: significant differences with GC;[D]: significant differences with GD; HDL: high density lipoprotein; hs-CRP: high-sensitivity C-reactive protein; LDL: low density lipoprotein; TC: total cholesterol; VLDL: very low density lipoprotein. One-way ANOVA with Sidak's post hoc test with $p < 0.01$.

Supplementary Table 3. Sex-based differences in CAM, CRF and metabolic indices/variables.

Type	Variables	All (n = 127)	Female (n = 56)	Male (n = 71)	*p* (sex)
CAM and CRF	SE_{HP}	3.567 ± 0.407	3.638 ± 0.420	3.510 ± 0.389	0.067
	$0V_{HP\ (\%)}$	20.420 ± 12.845	17.752 ± 11.975	22.524 ± 13.197	0.031
	$1V_{HP\ (\%)}$	46.016 ± 6.014	45.718 ± 5.998	46.252 ± 6.059	0.601
	$2LV_{HP\ (\%)}$	12.784 ± 6.345	14.595 ± 7.123	11.356 ± 5.282	**0.008**
	$2UV_{HP\ (\%)}$*	16.929 (12.205 - 28.150)	19.488 (12.992 - 28.740)	16.535 (11.417 - 25.394)	0.163
	$LF_{HP\ (ms2)}$	721.642 ± 862.322	754.441 ± 1104.352	695.772 ± 615.374	0.312
	$LF_{HP\ (nu)}$*	55.800 (39.091 - 73.081)	48.967 (39.825 - 72.143)	60.143 (39.091 - 74.419)	0.238
	$HF_{HP\ (ms2)}$	695.083 ± 911.758	690.371 ± 901.381	698.799 ± 926.245	0.886
	$HF_{HP\ (nu)}$	43.223 ± 22.014	45.445 ± 21.901	41.471 ± 22.100	0.314
	LF/HF_{HP}	2.558 ± 3.247	2.354 ± 3.353	2.718 ± 3.177	0.259
	SE_{SAP}	3.384 ± 0.308	3.412 ± 0.302	3.361 ± 0.313	0.361
	$0V_{SAP\ (\%)}$	32.063 ± 12.136	29.717 ± 10.929	33.914 ± 12.782	0.053
	$1V_{SAP\ (\%)}$	47.880 ± 5.267	48.967 ± 4.996	47.022 ± 5.351	0.034
	$2LV_{SAP\ (\%)}$	7.914 ± 4.994	8.732 ± 4.959	7.270 ± 4.962	0.062
	$2UV_{SAP\ (\%)}$	12.143 ± 6.966	12.584 ± 6.447	11.794 ± 7.377	0.256
	$LF_{SAP\ (ms2)}$	8.388 ± 12.613	6.537 ± 9.883	9.849 ± 14.308	0.150
	$LF_{SAP\ (nu)}$*	73.340 (58.308 - 88.243)	67.653 (55.999 - 82.313)	79.380 (64.092 - 90.426)	**0.006**
	$HF_{SAP\ (ms2)}$	1.681 ± 1.454	1.801 ± 1.591	1.587 ± 1.340	0.401
	$HF_{SAP\ (nu)}$ *	25.250 (11.757 - 40.169)	32.062 (16.883 - 41.679)	20.621 (9.221 - 35.066)	0.014
	$SEQ_{(\%)}$*	2.778 (1.587 - 4.762)	2.381 (1.190 - 4.117)	3.571 (1.984 - 5.159)	0.032
	$\alpha_{seq\ (ms \bullet mmHg-1)}$ *	13.104 (8.988 - 20.484)	13.873 (9.070 - 20.842)	12.649 (8.944 - 19.911)	0.877
	$\dot{V}O_{2PEAK\ (ml/kg/min)}$	31.431 ± 7.716	26.579 ± 5.916	35.259 ± 6.780	**< 0.001**
^1H NMR (mM)	2-Hydroxybutyrate	0.044 ± 0.016	0.041 ± 0.016	0.047 ± 0.016	0.019
	2-Hydroxyisovalerate	0.009 ± 0.004	0.007 ± 0.003	0.011 ± 0.004	**< 0.001**
	2-Oxoglutarate *	0.005 (0.003 - 0.007)	0.005 (0.003 - 0.006)	0.004 (0.003 - 0.007)	0.897
	2-Oxoisocaproate *	0.005 (0.004 - 0.006)	0.005 (0.004 - 0.005)	0.005 (0.004 - 0.006)	0.130
	3-Hydroxybutyrate *	0.037 (0.020 - 0.078)	0.032 (0.017 - 0.080)	0.039 (0.024 - 0.072)	0.340
	3-Hydroxyisobutyrate	0.016 ± 0.006	0.015 ± 0.004	0.018 ± 0.007	**0.002**
	3-Methyl-2-oxovalerate	0.005 ± 0.002	0.005 ± 0.002	0.005 ± 0.002	0.207
	Acetate *	0.059 ± 0.026	0.056 ± 0.021	0.062 ± 0.029	0.370
	Acetoacetate *	0.022 (0.014 - 0.036)	0.020 (0.014 - 0.031)	0.024 (0.015 - 0.038)	0.329
	Alanine *	0.444 (0.389 - 0.519)	0.425 (0.356 - 0.504)	0.457 (0.393 - 0.523)	0.084
	Anserine	0.019 ± 0.007	0.017 ± 0.006	0.020 ± 0.008	0.020
	Ascorbate	0.043 ± 0.016	0.045 ± 0.017	0.042 ± 0.015	0.294
	Asparagine *	0.056 (0.048 - 0.065)	0.055 (0.046 - 0.061)	0.057 (0.050 - 0.067)	0.120
	Aspartate	0.031 ± 0.009	0.031 ± 0.009	0.030 ± 0.009	0.652
	Betaine *	0.047 (0.040 - 0.058)	0.045 (0.038 - 0.050)	0.052 (0.044 - 0.059)	**0.001**
	Choline	0.011 ± 0.003	0.010 ± 0.002	0.012 ± 0.003	**0.005**
	Citrate *	0.068 (0.059 - 0.078)	0.066 (0.058 - 0.077)	0.068 (0.061 - 0.078)	0.756
	Creatine *	0.025 (0.014 - 0.041)	0.037 (0.025 - 0.045)	0.017 (0.011 - 0.027)	**< 0.001**
	Creatinine	0.087 ± 0.020	0.071 ± 0.013	0.099 ± 0.015	**< 0.001**
	Dimethyl sulfone *	0.008 (0.006 - 0.011)	0.008 (0.006 - 0.013)	0.008 (0.006 - 0.011)	0.657
	Dimethylamine *	0.004 (0.003 - 0.004)	0.003 (0.003 - 0.004)	0.004 (0.003 - 0.004)	0.016
	Ethanol	0.195 ± 0.157	0.190 ± 0.118	0.198 ± 0.183	0.680
	Formate	0.046 ± 0.012	0.047 ± 0.012	0.046 ± 0.011	0.534
	Glucose *	3.481 (3.185 - 3.811)	3.416 (3.056 - 3.744)	3.556 (3.267 - 3.811)	0.087
	Glutamine	0.490 ± 0.067	0.482 ± 0.075	0.496 ± 0.060	0.169
	Glycerol	1.570 ± 1.234	1.250 ± 0.848	1.823 ± 1.425	0.017
	Glycine *	0.320 (0.267 - 0.377)	0.343 (0.293 - 0.428)	0.313 (0.256 - 0.359)	**0.004**
	Histidine	0.087 ± 0.016	0.082 ± 0.017	0.090 ± 0.015	**0.006**
	Hypoxanthine	0.013 ± 0.004	0.013 ± 0.004	0.013 ± 0.004	0.500
	Isoleucine	0.073 ± 0.016	0.065 ± 0.011	0.078 ± 0.018	**< 0.001**
	Lactate	3.120 ± 0.862	2.852 ± 0.865	3.331 ± 0.805	**0.001**
	Leucine	0.099 ± 0.020	0.089 ± 0.018	0.107 ± 0.019	**< 0.001**
	Lysine	0.146 ± 0.023	0.140 ± 0.023	0.151 ± 0.023	**0.006**
	Methionine	0.030 ± 0.005	0.028 ± 0.005	0.031 ± 0.005	**< 0.001**

Supplementary Table 3. Continued

	Variables	All (n = 127)	Female (n = 56)	Male (n = 71)	*p* (sex)
	N,N-Dimethylglycine *	0.003 (0.003 - 0.004)	0.003 (0.002 - 0.003)	0.004 (0.003 - 0.004)	**< 0.001**
	O-Acetylcarnitine	0.005 ± 0.002	0.004 ± 0.002	0.005 ± 0.002	0.390
	Ornithine *	0.068 (0.060 - 0.078)	0.066 (0.054 - 0.076)	0.070 (0.064 - 0.081)	0.045
	Phenylalanine	0.073 ± 0.010	0.069 ± 0.008	0.076 ± 0.010	**< 0.001**
	Proline *	0.233 (0.202 - 0.281)	0.207 (0.176 - 0.245)	0.248 (0.222 - 0.296)	**< 0.001**
	Pyruvate	0.065 ± 0.033	0.064 ± 0.030	0.065 ± 0.036	0.853
	Sarcosine *	0.002 (0.002 - 0.003)	0.002 (0.002 - 0.003)	0.003 (0.002 - 0.003)	0.121
	Serine	0.123 ± 0.022	0.128 ± 0.022	0.118 ± 0.021	0.017
	Succinate *	0.008 (0.007 - 0.010)	0.008 (0.007 - 0.009)	0.008 (0.008 - 0.010)	0.075
	Threonine	0.173 ± 0.035	0.173 ± 0.039	0.172 ± 0.031	0.931
	Trimethylamine *	0.002 (0.001 - 0.003)	0.001 (0.001 - 0.002)	0.002 (0.002 - 0.003)	**< 0.001**
	Tyrosine *	0.088 (0.078 - 0.100)	0.086 (0.076 - 0.097)	0.091 (0.080 - 0.100)	0.202
	Valine	0.253 ± 0.050	0.236 ± 0.043	0.267 ± 0.050	**< 0.001**
LC-HRMS (a.u.)	158.964m/z	0.906 ± 0.197	0.909 ± 0.225	0.903 ± 0.174	0.758
	141.958m/z *	1.088 (1.033 - 1.155)	1.113 (1.031 - 1.158)	1.081 (1.034 - 1.131)	0.531
	181.950m/z	0.998 ± 0.098	0.986 ± 0.097	1.008 ± 0.098	0.167
	281.101m/z	1.017 ± 0.214	1.053 ± 0.234	0.989 ± 0.194	0.120
	1433.834m/z *	1.021 (0.969 - 1.099)	1.011 (0.968 - 1.089)	1.033 (0.970 - 1.107)	0.659
	1398.798m/z	0.988 ± 0.098	0.984 ± 0.091	0.992 ± 0.103	0.735
	337.167m/z	0.968 ± 0.147	0.979 ± 0.154	0.960 ± 0.141	0.470
	1456.841m/z *	0.965 (0.906 - 1.041)	0.939 (0.877 - 1.015)	0.986 (0.925 - 1.090)	**0.008**
	1514.883m/z *	0.941 (0.864 - 1.018)	0.942 (0.877 - 1.002)	0.938 (0.862 - 1.033)	0.934
	1572.925m/z	1.001 ± 0.159	1.006 ± 0.141	0.998 ± 0.173	0.883
	1630.967m/z	0.964 ± 0.130	0.948 ± 0.120	0.977 ± 0.137	0.220
	384.347m/z	0.949 ± 0.148	0.937 ± 0.164	0.959 ± 0.134	0.510
	1689.009m/z *	1.031 (0.872 - 1.116)	1.000 (0.842 - 1.084)	1.077 (0.911 - 1.120)	0.042
	1546.006m/z *	1.015 (0.948 - 1.086)	0.986 (0.942 - 1.074)	1.034 (0.954 - 1.088)	0.188
	1805.093m/z *	0.980 (0.950 - 1.029)	0.970 (0.939 - 1.015)	0.988 (0.959 - 1.032)	0.102
	158.978m/z *	1.032 (0.826 - 1.251)	0.953 (0.733 - 1.177)	1.089 (0.855 - 1.271)	0.188
	112.986m/z *	0.961 (0.898 - 1.095)	0.961 (0.878 - 1.077)	0.969 (0.904 - 1.113)	0.550
	199.805m/z	1.117 ± 0.350	1.116 ± 0.267	1.119 ± 0.406	0.633
	111.009m/z	0.949 ± 0.369	0.943 ± 0.406	0.955 ± 0.339	0.658
	197.808m/z	1.118 ± 0.329	1.119 ± 0.273	1.117 ± 0.369	0.488
	180.067m/z *	0.956 (0.810 - 1.129)	0.961 (0.799 - 1.097)	0.956 (0.843 - 1.152)	0.518
	130.087m/z *	0.934 (0.817 - 1.076)	0.827 (0.732 - 0.940)	0.992 (0.910 - 1.109)	**< 0.001**
	103.040m/z	0.990 ± 0.610	0.912 ± 0.464	1.051 ± 0.701	0.152
	164.072m/z *	0.912 (0.845 - 1.040)	0.866 (0.804 - 0.972)	0.963 (0.885 - 1.063)	**0.002**
	117.056m/z *	0.850 (0.637 - 1.125)	0.649 (0.531 - 0.770)	1.016 (0.862 - 1.291)	**< 0.001**
	145.051m/z *	1.386 (1.134 - 1.806)	1.402 (1.067 - 1.887)	1.302 (1.148 - 1.713)	0.819
	203.083m/z *	0.931 (0.844 - 1.065)	0.889 (0.802 - 0.954)	1.002 (0.888 - 1.095)	**< 0.001**
	181.050m/z	1.020 ± 0.542	0.786 ± 0.349	1.204 ± 0.596	**< 0.001**
	172.098m/z *	0.229 (0.082 - 1.017)	0.159 (0.077 - 0.751)	0.302 (0.089 - 1.125)	0.188
	178.051m/z	0.939 ± 0.803	1.021 ± 1.067	0.874 ± 0.507	0.628
	263.103m/z	1.015 ± 0.783	1.014 ± 0.917	1.016 ± 0.666	0.485
	173.082m/z	0.753 ± 0.399	0.711 ± 0.421	0.785 ± 0.380	0.179
	129.056m/z	1.007 ± 0.367	0.885 ± 0.248	1.104 ± 0.416	**< 0.001**
	201.113m/z *	0.835 (0.592 - 1.055)	0.805 (0.584 - 0.980)	0.851 (0.600 - 1.141)	0.174
	212.002m/z *	0.983 (0.641 - 1.259)	0.867 (0.604 - 1.192)	1.075 (0.737 - 1.281)	0.119
	187.007m/z	0.961 ± 0.786	1.075 ± 0.990	0.871 ± 0.567	0.346
	644.959m/z	0.960 ± 0.160	0.953 ± 0.176	0.966 ± 0.148	0.771

* Mann-Whitney test (data presented as median and interquartile range). Data are mean ± standard deviation. 0V: no variation 1V: one variation; 2LV: two like variations; 2UV: two unlike variations; α_{seq}: baroreflex sensitivity (BRS); HF: high frequency band; HF_{nu}: HF in normalized unites; HP: heart period; LF: low frequency band; LF_{nu}: LF in normalized unites; SAP: systolic arterial pressure; SE: Shannon entropy; SEQ: percentage of HP-SAP patterns of baroreflex origin; $\dot{V}O_{2PEAK}$: peak oxygen consumption. Independent t-test with $p < 0.01$

Supplementary Table 4. Metabolic variables detected and measured by ^{1}H NMR and LC-HRMS analytical techniques for each group evaluated for female individuals.

Molecules	GA (n = 16)	GB (n = 11)	GC (n = 14)	GD (n = 15)	p
***^{1}H NMR* (mM)**					
2-Hydroxybutyrate	0.0459 ± 0.0245	0.0378 ± 0.0085	0.0393 ± 0.0086	0.0401 ± 0.0124	0.969
2-Hydroxyisovalerate	0.0066 ± 0.0026	0.0077 ± 0.0035	0.0061 ± 0.0017	0.0078 ± 0.0043	0.933
2-Oxoglutarate	0.0054 ± 0.0035	0.0047 ± 0.0023	0.0045 ± 0.0022	0.0042 ± 0.0021	0.895
2-Oxoisocaproate	0.0044 ± 0.0010	0.0045 ± 0.0014	0.0051 ± 0.0013	0.0045 ± 0.0016	0.508
3-Hydroxybutyrate	0.0556 ± 0.0591	0.0433 ± 0.0383	0.0497 ± 0.0484	0.0612 ± 0.0453	0.715
3-Hydroxyisobutyrate	0.0151 ± 0.0040	0.0145 ± 0.0039	0.0142 ± 0.0032	0.0145 ± 0.0056	0.878
3-Methyl-2-oxovalerate	0.0045 ± 0.0022	0.0046 ± 0.0011	0.0049 ± 0.0015	0.0052 ± 0.0019	0.745
Acetate*	0.0652 (0.0421 - 0.073)	0.0472 (0.0352 - 0.0582)	0.0606 (0.0406 - 0.0717)	0.0472 (0.0414 - 0.0566)	0.403
Acetoacetate	0.0296 ± 0.0256	0.0235 ± 0.0173	0.0260 ± 0.0183	0.0329 ± 0.0239	0.650
Alanine	0.4457 ± 0.1053	0.4350 ± 0.0904	0.4618 ± 0.0999	0.4093 ± 0.0870	0.552
Anserine	0.0182 ± 0.0074	0.0175 ± 0.0069	0.0176 ± 0.0042	0.0145 ± 0.0030	0.542
Ascorbate	0.0517 ± 0.0145	0.0403 ± 0.0226	0.0498 ± 0.0124	0.0368 ± 0.0151	0.123
Asparagine	0.0549 ± 0.0129	0.0536 ± 0.0063	0.0581 ± 0.0149	0.0553 ± 0.0132	0.578
Aspartate	0.0301 ± 0.0071	0.0277 ± 0.0101	0.0343 ± 0.0112	0.0321 ± 0.0094	0.592
Betaine	0.0476 ± 0.0090	0.0434 ± 0.0113	0.0455 ± 0.0112	0.0418 ± 0.0122	0.376
Choline	0.0111 ± 0.0023	0.0091 ± 0.0018	0.0111 ± 0.0028	0.0094 ± 0.0018	0.108
Citrate	0.0749 ± 0.0313	0.0650 ± 0.0141	0.0732 ± 0.0120	0.0655 ± 0.0117	0.867
Creatine	0.0393 ± 0.0202	0.0324 ± 0.0260	0.0335 ± 0.0186	0.0421 ± 0.0123	0.266
Creatinine	0.0695 ± 0.0123	0.0691 ± 0.0149	0.0770 ± 0.0135	0.0681 ± 0.0116	0.302
Dimethyl sulfone*	0.0076 (0.0058 - 0.0126)	0.0076 (0.006 - 0.0090)	0.0104 (0.0096 - 0.0139)	0.0068 (0.0050 - 0.0138)	0.217
Dimethylamine	0.0035 ± 0.0008	0.0030 ± 0.0006	0.0037 ± 0.0007	0.0033 ± 0.0008	0.344
Ethanol	0.2023 ± 0.1123	0.1832 ± 0.1151	0.1839 ± 0.0999	0.1886 ± 0.1486	0.899
Formate	0.0483 ± 0.0166	0.0455 ± 0.0107	0.0459 ± 0.0112	0.0468 ± 0.0097	0.970
Glucose	3.4854 ± 0.4853	3.1453 ± 0.3958	3.6279 ± 0.5402	3.4009 ± 0.7208	0.551
Glutamine	0.4942 ± 0.0695	0.4684 ± 0.0858	0.4760 ± 0.0810	0.4863 ± 0.0728	0.762
Glycerol	1.2340 ± 0.4999	0.9173 ± 0.4633	1.6635 ± 1.4426	1.1257 ± 0.4460	0.306
Glycine*	0.3400 (0.2944 - 0.4067)	0.3248 (0.2758 - 0.4112)	0.3576 (0.3417 - 0.4120)	0.3356 (0.2200 - 0.5060)	0.707
Histidine	0.0849 ± 0.0175	0.0748 ± 0.0223	0.0855 ± 0.0102	0.0819 ± 0.0180	0.062
Hypoxanthine	0.0141 ± 0.0034	0.0124 ± 0.0045	0.0127 ± 0.0042	0.0130 ± 0.0037	0.382
Isoleucine	0.0667 ± 0.0101	0.0673 ± 0.0119	0.0630 ± 0.0142	0.0651 ± 0.0097	0.863
Lactate	2.9434 ± 0.8564	2.6888 ± 0.8507	3.2788 ± 0.9454	2.4756 ± 0.6670	0.152
Leucine	0.0924 ± 0.0169	0.0879 ± 0.0182	0.0863 ± 0.0195	0.0881 ± 0.0173	0.740
Lysine	0.1454 ± 0.0198	0.1390 ± 0.0288	0.1370 ± 0.0168	0.1370 ± 0.0269	0.658
Methionine	0.0282 ± 0.0058	0.0277 ± 0.0044	0.0283 ± 0.0047	0.0281 ± 0.0045	0.466
N,N-Dimethylglycine*	0.0032 (0.0028 - 0.0037)	0.0028 (0.0022 - 0.0032)	0.0028 (0.0025 - 0.0044)	0.0024 (0.0022 - 0.0028)	0.014
O-Acetylcarnitine	0.0049 ± 0.0026	0.0038 ± 0.0019	0.0043 ± 0.0015	0.0047 ± 0.0022	0.850
Ornithine	0.0693 ± 0.0139	0.0576 ± 0.0094	0.0768 ± 0.0222	0.0635 ± 0.0155	0.325
Phenylalanine	0.0700 ± 0.0087	0.0644 ± 0.0051	0.0706 ± 0.0098	0.0682 ± 0.0087	0.302
Proline*	0.2142 (0.1798 - 0.2257)	0.1752 (0.1686 - 0.2228)	0.2136 (0.2018 - 0.2420)	0.2016 (0.1766 - 0.2648)	0.574
Pyruvate	0.0683 ± 0.0320	0.0618 ± 0.0251	0.0688 ± 0.0366	0.0549 ± 0.0251	0.405
Sarcosine*	0.0026 (0.0023 - 0.0033)	0.0024 (0.0018 - 0.0028)	0.0028 (0.0021 - 0.0035)	0.0020 (0.0016 - 0.0022)	0.047
Serine	0.1296 ± 0.0211	0.1238 ± 0.0292	0.1243 ± 0.0188	0.1319 ± 0.0216	0.682
Succinate	0.0078 ± 0.0020	0.0075 ± 0.0010	0.0089 ± 0.0022	0.0080 ± 0.0014	0.471
Threonine*	0.1708 (0.1523 - 0.1832)	0.1860 (0.1676 - 0.2138)	0.1558 (0.1408 - 0.1894)	0.1640 (0.1556 - 0.1870)	0.363
Trimethylamine	0.0014 ± 0.0005	0.0014 ± 0.0008	0.0016 ± 0.0009	0.0013 ± 0.0005	0.798
Tyrosine	0.0926 ± 0.0165	0.0826 ± 0.0111	0.0887 ± 0.0181	0.0841 ± 0.0163	0.347
Valine*	0.2484 (0.2147 - 0.2733)	0.2516 (0.2150 - 0.2676)	0.2084 (0.1920 - 0.2447)	0.2308 (0.2188 - 0.2508)	0.303
***LC-HRMS* (a.u.)**	**GA (n = 16)**	**GB (n = 11)**	**GC (n = 14)**	**GD (n = 15)**	***p***
158.964m/z	0.9708 ± 0.2475	0.8798 ± 0.1259	0.8686 ± 0.2010	0.9039 ± 0.2803	0.659
141.958m/z	1.2322 ± 0.3545	1.1595 ± 0.1377	1.0707 ± 0.1267	1.0310 ± 0.1228	0.066
181.950m/z	0.9553 ± 0.0769	1.0288 ± 0.1031	1.0103 ± 0.0812	0.9632 ± 0.1155	0.091
281.101m/z	1.0818 ± 0.2602	0.9518 ± 0.1468	1.0922 ± 0.2746	1.0598 ± 0.2155	0.521
1433.834m/z*	1.0461 (1.0022 - 1.0927)	1.0531 (1.0046 - 1.1004)	0.9821 (0.9263 - 1.0658)	0.9832 (0.9581 - 1.0186)	0.189
1398.798m/z	1.0008 ± 0.0955	0.9896 ± 0.0845	0.9801 ± 0.0774	0.9642 ± 0.1060	0.573
337.167m/z	1.0356 ± 0.1728	0.9496 ± 0.1055	0.9473 ± 0.1657	0.9689 ± 0.1493	0.357
1456.841m/z	0.9079 ± 0.1294	1.0236 ± 0.1200	0.9163 ± 0.1290	0.9039 ± 0.1600	0.176
1514.883m/z	0.9487 ± 0.2129	0.9839 ± 0.1394	0.8950 ± 0.0841	0.9682 ± 0.1442	0.869

Supplementary Table 4. Continued

1572.925m/z	1.0032 ± 0.1110	1.0536 ± 0.1325	1.0113 ± 0.1680	0.9705 ± 0.1517	0.480
1630.967m/z	0.9549 ± 0.0971	0.9768 ± 0.1091	0.8816 ± 0.1314	0.9828 ± 0.1262	0.413
384.347m/z*	1.0299 (0.9629 - 1.1088)	0.9259 (0.8917 - 1.0641)	0.8721 (0.8476 - 0.9403)	0.9377 (0.8704 - 1.0135)	0.011
1689.009m/z	0.9243 ± 0.3168	0.9169 ± 0.2180	0.8315 ± 0.3394	0.9511 ± 0.2843	0.806
1546.006m/z*	0.9502 (0.9296 - 1.0765)	1.0197 (0.9218 - 1.1093)	0.9738 (0.9458 - 1.0079)	0.9949 (0.9790 - 1.0897)	0.394
1805.093m/z	0.9717 ± 0.0559	1.0080 ± 0.0719	0.9663 ± 0.0589	0.9790 ± 0.0647	0.759
158.978m/z	1.0496 ± 0.2843	0.8470 ± 0.2901	0.9478 ± 0.3165	1.1156 ± 0.3342	0.092
112.986m/z	1.0620 ± 0.1805	0.9372 ± 0.0992	1.0199 ± 0.1633	0.9860 ± 0.1735	0.351
199.805m/z	1.0491 ± 0.2124	1.0701 ± 0.2519	1.2017 ± 0.2807	1.1402 ± 0.3149	0.185
111.009m/z	1.0356 ± 0.4804	0.8154 ± 0.3241	0.9058 ± 0.3539	0.9712 ± 0.4291	0.613
197.808m/z	1.1014 ± 0.3003	1.0397 ± 0.1674	1.1981 ± 0.2614	1.1220 ± 0.3195	0.223
180.067m/z	1.1503 ± 0.5478	0.8651 ± 0.1226	0.9056 ± 0.2070	1.1945 ± 0.8318	0.355
130.087m/z	1.0505 ± 0.6193	0.8359 ± 0.1503	0.8609 ± 0.2219	1.0833 ± 0.7612	0.721
103.040m/z	0.9619 ± 0.4382	0.7416 ± 0.1676	0.8469 ± 0.3501	1.0455 ± 0.6771	0.631
164.072m/z	1.0225 ± 0.3864	0.8245 ± 0.1328	0.9482 ± 0.2889	1.0583 ± 0.5917	0.448
117.056m/z	0.7437 ± 0.3091	0.7326 ± 0.2598	0.5650 ± 0.1657	0.8588 ± 0.4740	0.271
145.051m/z	1.5373 ± 0.4804	1.2839 ± 0.5550	2.0736 ± 1.7438	3.9388 ± 6.1103	0.090
203.083m/z*	0.8876 (0.8024 - 0.9378)	0.8619 (0.7659 - 0.9252)	0.8722 (0.8215 - 0.9353)	0.9489 (0.8084 - 0.9934)	0.765
181.050m/z	0.8283 ± 0.4164	0.7648 ± 0.3346	0.7219 ± 0.1892	0.8168 ± 0.4154	0.896
172.098m/z	0.8908 ± 2.9018	0.6463 ± 1.1245	1.0976 ± 1.8792	0.8544 ± 1.0675	0.275
178.051m/z	0.9838 ± 0.7606	1.3796 ± 1.8625	1.1970 ± 0.9391	0.6345 ± 0.5107	0.486
263.103m/z	1.0262 ± 0.7865	0.8174 ± 0.6043	1.0954 ± 0.5864	1.0675 ± 1.4217	0.941
173.082m/z*	0.5107 (0.4299 - 0.6249)	0.5822 (0.5008 - 0.6586)	0.876 (0.6193 - 1.3738)	0.5163 (0.4686 - 0.8063)	0.063
129.056m/z	0.8854 ± 0.2384	0.8342 ± 0.1175	0.8048 ± 0.1944	0.9963 ± 0.3368	0.434
201.113m/z	0.9208 ± 0.5455	0.7709 ± 0.1950	1.0212 ± 0.2644$^{\mathbf{D}}$	0.5671 ± 0.3833^{C}	**0.008**
212.002m/z	1.0634 ± 0.7355	0.9585 ± 0.2973	0.9052 ± 0.4663	0.9738 ± 0.7671	0.658
187.007m/z	1.1108 ± 0.8088	0.9100 ± 0.4789	1.1752 ± 0.7211	1.0655 ± 1.5725	0.730
644.959m/z	0.9557 ± 0.1793	0.9077 ± 0.2157	0.9477 ± 0.1828	0.9880 ± 0.1412	0.724

* Kruskal-Wallis test (data presented as median and interquartile range). Data are mean ± standard deviation. ^{A}: significant differences with GA; ^{B}: significant differences with GB; ^{C}: significant differences with GC;^{D}: significant differences with GD; GA: group A; GB: group B; GC: group C; GD: group D. One-way ANOVA with Sidak's post hoc test controlled for age and $p < 0.01$.

Supplementary Table 5. Metabolic variables detected and measured by ^{1}H NMR and LC-HRMS analytical techniques for each group evaluated for male individuals.

Molecules	GA (n = 17)	GB (n = 16)	GC (n = 19)	GD (n = 19)	*p*
***^{1}H NMR* (mM)**					
2-Hydroxybutyrate	0.0511 ± 0.0206	0.0411 ± 0.0125	0.0493 ± 0.0123	0.0447 ± 0.0155	0.134
2-Hydroxyisovalerate	0.0097 ± 0.0038	0.0107 ± 0.0035	0.0109 ± 0.0055	0.0111 ± 0.0047	0.765
2-Oxoglutarate	0.0061 ± 0.0041	0.0048 ± 0.0025	0.0048 ± 0.0027	0.0036 ± 0.0024	0.149
2-Oxoisocaproate	0.0046 ± 0.0019	0.0049 ± 0.0012	0.0053 ± 0.0013	0.0050 ± 0.0013	0.539
3-Hydroxybutyrate*	0.0468 (0.0304 - 0.0992)	0.0404 (0.0192 - 0.0537)	0.0364 (0.0244 - 0.0756)	0.0372 (0.0218 - 0.0646)	0.490
3-Hydroxyisobutyrate	0.0206 ± 0.0084	0.0165 ± 0.0044	0.0183 ± 0.0074	0.0167 ± 0.0053	0.053
3-Methyl-2-oxovalerate	0.0048 ± 0.0021	0.0053 ± 0.0018	0.0052 ± 0.0022	0.0056 ± 0.0024	0.558
Acetate	0.0687 ± 0.0321	0.0551 ± 0.0280	0.0630 ± 0.0263	0.0604 ± 0.0308	0.365
Acetoacetate	0.0425 ± 0.0370	0.0301 ± 0.0282	0.0298 ± 0.0236	0.0361 ± 0.0429	0.159
Alanine	0.4714 ± 0.1210	0.4617 ± 0.1054	0.4623 ± 0.0813	0.4697 ± 0.0959	0.980
Anserine	0.0192 ± 0.0059	0.0191 ± 0.0081	0.0188 ± 0.0059	0.0221 ± 0.0102	0.660
Ascorbate	0.0408 ± 0.0147	0.0373 ± 0.0134	0.0413 ± 0.0180	0.0463 ± 0.0142	0.366
Asparagine	0.0643 ± 0.0134	0.0568 ± 0.0142	0.0561 ± 0.0106	0.0576 ± 0.0110	0.213
Aspartate	0.0342 ± 0.0082	0.0276 ± 0.0092	0.0300 ± 0.0071	0.0300 ± 0.0104	0.482
Betaine	0.0552 ± 0.0111	0.0516 ± 0.0120	0.0479 ± 0.0119	0.0544 ± 0.0176	0.276
Choline	0.0122 ± 0.0027	0.0112 ± 0.0030	0.0114 ± 0.0022	0.0114 ± 0.0028	0.832
Citrate	0.0725 ± 0.0182	0.0689 ± 0.0176	0.0676 ± 0.0138	0.0647 ± 0.0146	0.507
Creatine	0.0284 ± 0.0218	0.0139 ± 0.0076	0.0245 ± 0.0153	0.0210 ± 0.0152	0.018
Creatinine	0.1009 ± 0.0168	0.0982 ± 0.0156	0.0948 ± 0.0151	0.1026 ± 0.0142	0.303
Dimethyl sulfone	0.0121 ± 0.0062	0.0074 ± 0.0027	0.0086 ± 0.0030	0.0080 ± 0.0035	0.037
Dimethylamine	0.0042 ± 0.0010	0.0034 ± 0.0004	0.0036 ± 0.0006	0.0037 ± 0.0007	0.041
Ethanol	0.1744 ± 0.1215	0.1893 ± 0.1241	0.1657 ± 0.0685	0.2596 ± 0.3076	0.620
Formate	0.0482 ± 0.0091	0.0438 ± 0.0162	0.0453 ± 0.0092	0.0449 ± 0.0107	0.482
Glucose	3.5880 ± 0.4633	3.5195 ± 0.4966	3.8145 ± 0.7196	3.5074 ± 0.3161	0.399
Glutamine	0.5012 ± 0.0533	0.4900 ± 0.0670	0.4954 ± 0.0620	0.4957 ± 0.0627	0.689
Glycerol	1.5637 ± 1.1554	1.9615 ± 1.5846	1.9972 ± 1.5734	1.7632 ± 1.4218	0.820
Glycine*	0.3068 (0.2656 - 0.3472)	0.3092 (0.2707 - 0.3569)	0.3164 (0.2446 - 0.3528)	0.3152 (0.2610 - 0.3654)	0.929
Histidine	0.0945 ± 0.0152	0.0871 ± 0.0149	0.0882 ± 0.0160	0.0901 ± 0.0144	0.527
Hypoxanthine	0.0141 ± 0.0033	0.0129 ± 0.0039	0.0134 ± 0.0033	0.0135 ± 0.0040	0.694
Isoleucine	0.0776 ± 0.0210	0.0738 ± 0.0159	0.0823 ± 0.0197	0.0789 ± 0.0130	0.107
Lactate	3.5844 ± 0.9705	3.0582 ± 0.7771	3.2810 ± 0.7567	3.3826 ± 0.6887	0.371
Leucine*	0.0980 (0.0852 - 0.1180)	0.1068 (0.0941 - 0.1119)	0.1072 (0.0990 - 0.1190)	0.1072 (0.0976 - 0.1176)	0.687
Lysine	0.1499 ± 0.0179	0.1380 ± 0.0192	0.1609 ± 0.0243	0.1531 ± 0.0238	0.043
Methionine	0.0317 ± 0.0046	0.0298 ± 0.0033	0.0311 ± 0.0041	0.0324 ± 0.0062	0.063
N,N-Dimethylglycine	0.0046 ± 0.0017	0.0036 ± 0.0011	0.0036 ± 0.0009	0.0037 ± 0.0007	0.018
O-Acetylcarnitine	0.0057 ± 0.0022	0.0045 ± 0.0016	0.0043 ± 0.0015	0.0044 ± 0.0018	0.088
Ornithine	0.0796 ± 0.0127^{B}	0.0612 ± 0.0148^{A}	0.0707 ± 0.0129	0.0754 ± 0.0141	**0.008**
Phenylalanine	0.0785 ± 0.0109	0.0717 ± 0.0080	0.0771 ± 0.0097	0.0780 ± 0.0121	0.182
Proline	0.2699 ± 0.0404	0.2760 ± 0.0904	0.2640 ± 0.0585	0.2451 ± 0.0590	0.273
Pyruvate	0.0814 ± 0.0531	0.0692 ± 0.0269	0.0566 ± 0.0324	0.0568 ± 0.0208	0.299
Sarcosine*	0.0028 (0.0024 - 0.0028)	0.0026 (0.0024 - 0.0029)	0.0028 (0.0022 - 0.0028)	0.0024 (0.0020 - 0.0028)	0.246
Serine	0.1239 ± 0.0181	0.1108 ± 0.0235	0.1213 ± 0.0140	0.1172 ± 0.0253	0.063
Succinate	0.0086 ± 0.0021	0.0081 ± 0.0024	0.0079 ± 0.0024	0.0091 ± 0.0015	0.433
Threonine	0.1848 ± 0.0342	0.1709 ± 0.0261	0.1671 ± 0.0327	0.1669 ± 0.0303	0.209
Trimethylamine*	0.0028 (0.002 - 0.0032)	0.0026 (0.0016 - 0.0032)	0.002 (0.0016 - 0.0032)	0.0024 (0.002 - 0.0028)	0.850
Tyrosine	0.0955 ± 0.0197	0.0847 ± 0.0091	0.0928 ± 0.0155	0.0941 ± 0.0213	0.144
Valine	0.2669 ± 0.0661	0.2577 ± 0.0461	0.2804 ± 0.0471	0.2611 ± 0.0405	0.119
***LC-HRMS* (a.u.)**	**GA (n = 16)**	**GB (n = 11)**	**GC (n = 14)**	**GD (n = 15)**	***p***
158.964m/z	0.9869 ± 0.2084	0.8237 ± 0.1439	0.8587 ± 0.1255	0.9395 ± 0.1737	0.015
141.958m/z*	1.0709 (1.0312 - 1.1146)	1.0651 (1.0394 - 1.1673)	1.0859 (1.0543 - 1.1681)	1.0782 (1.0268 - 1.1104)	0.993
181.950m/z	0.9841 ± 0.1119	1.0303 ± 0.0701	1.0000 ± 0.1197	1.0178 ± 0.0827	0.479
281.101m/z	0.9863 ± 0.1917	0.9950 ± 0.1529	0.9634 ± 0.1916	1.0133 ± 0.2375	0.684
1433.834m/z	1.0316 ± 0.1219	0.9948 ± 0.1088	1.1316 ± 0.3460	1.0436 ± 0.0972	0.083
1398.798m/z	0.9959 ± 0.0880^{B}	0.9197 ± 0.0627^{ACD}	1.0171 ± 0.1100^{B}	1.0247 ± 0.1135^{B}	**0.002**
337.167m/z	1.0022 ± 0.0998	0.9153 ± 0.1358	0.9373 ± 0.1633	0.9815 ± 0.1496	0.156
1456.841m/z*	0.9707 (0.9542 - 1.0491)	0.9667 (0.9226 - 1.0336)	1.0366 (0.9430 - 1.1504)	0.9807 (0.8899 - 1.095)	0.451
1514.883m/z*	0.9160 (0.8627 - 1.0785)	0.8704 (0.8281 - 0.9999)	0.9901 (0.9709 - 1.0972)	0.9282 (0.8427 - 0.9936)	0.048

Supplementary Table 5. Continued

1572.925m/z	1.0548 ± 0.1337	0.9595 ± 0.1056	0.9784 ± 0.2574	0.9973 ± 0.1426	0.279
1630.967m/z	1.0152 ± 0.1428	0.9403 ± 0.1357	0.9734 ± 0.1373	0.9775 ± 0.1354	0.604
384.347m/z	0.9757 ± 0.1197	0.9281 ± 0.1750	0.9711 ± 0.1462	0.9583 ± 0.0952	0.971
1689.009m/z	1.1190 ± 0.1328	0.9306 ± 0.2564	1.0042 ± 0.1754	0.9801 ± 0.2077	0.011
1546.006m/z	1.0229 ± 0.0899	0.9966 ± 0.0761	1.0572 ± 0.0845	1.0281 ± 0.0993	0.528
1805.093m/z*	0.9856 (0.9556 - 1.051)	0.9697 (0.9471 - 1.0183)	1.0008 (0.9624 - 1.0205)	0.9985 (0.9714 - 1.0497)	0.757
158.978m/z	1.0072 ± 0.2863	0.9924 ± 0.2728	1.1409 ± 0.2383	1.0717 ± 0.2685	0.443
112.986m/z	0.9740 ± 0.0972	1.0080 ± 0.1049	1.0386 ± 0.1446	0.9978 ± 0.1403	0.553
199.805m/z	1.0321 ± 0.1956	1.2050 ± 0.6012	1.1757 ± 0.5046	1.0659 ± 0.1754	0.461
111.009m/z	1.0207 ± 0.3360	0.9174 ± 0.3402	1.0096 ± 0.3587	0.8716 ± 0.3264	0.606
197.808m/z	1.0278 ± 0.1770	1.1819 ± 0.4805	1.2194 ± 0.5080	1.0387 ± 0.1539	0.341
180.067m/z	1.0106 ± 0.2812	1.2243 ± 1.0868	1.6288 ± 1.9236	0.9358 ± 0.1924	0.295
130.087m/z	0.9946 ± 0.3041	1.3082 ± 1.2155	1.3475 ± 0.8625	0.9970 ± 0.1470	0.033
103.040m/z	1.0456 ± 0.4161	0.9650 ± 0.6828	1.3387 ± 1.0667	0.8408 ± 0.2992	0.107
164.072m/z*	0.9744 (0.8858 - 1.0096)	0.9242 (0.8945 - 0.9769)	1.0153 (0.9473 - 1.0626)	0.8901 (0.8493 - 1.1396)	0.439
117.056m/z	1.0269 ± 0.6320	1.3147 ± 0.9191	1.3993 ± 0.7170	1.0273 ± 0.4398	0.066
145.051m/z	1.5022 ± 0.4524	1.6446 ± 1.1787	3.7594 ± 5.9709	2.6265 ± 2.8995	0.797
203.083m/z	1.0169 ± 0.2593	1.0848 ± 0.4072	1.1762 ± 0.4758	0.9776 ± 0.1676	0.236
181.050m/z	1.1113 ± 0.4793	1.2631 ± 0.5331	1.3253 ± 0.9053	1.1143 ± 0.2886	0.505
172.098m/z*	0.4498 (0.1040 - 1.1651)	0.3587 (0.1824 - 1.6205)	0.4225 (0.1744 - 2.3423)	0.1427 (0.0797 - 0.3077)	0.044
178.051m/z	0.8374 ± 0.4170	0.6016 ± 0.2145	1.0756 ± 0.6898	0.9327 ± 0.4675	0.566
263.103m/z	1.1753 ± 0.8720	0.7930 ± 0.4326	1.1221 ± 0.6974	0.9550 ± 0.5699	0.832
173.082m/z	0.8943 ± 0.3864	0.8161 ± 0.3745	0.8364 ± 0.4274	0.6111 ± 0.2894	0.047
129.056m/z	1.1038 ± 0.2868	1.1277 ± 0.4335	1.2047 ± 0.6005	0.9826 ± 0.2386	0.198
201.113m/z*	0.8351 (0.7416 - 1.2602)	0.863 (0.561 - 1.1696)	0.9319 (0.5381 - 1.4152)	0.7905 (0.7636 - 0.9842)	0.986
212.002m/z	1.0934 ± 0.4938	0.9884 ± 0.3921	1.4740 ± 1.4018	1.0209 ± 0.3997	0.832
187.007m/z	0.8766 ± 0.6461	0.8449 ± 0.6335	0.9988 ± 0.5735	0.7593 ± 0.4288	0.593
644.959m/z	1.0393 ± 0.1538	0.9729 ± 0.1472	0.9758 ± 0.0884	0.8834 ± 0.1609	0.011

* Kruskal-Wallis test (data presented as median and interquartile range). Data are mean ± standard deviation. ^{A}: significant differences with GA; ^{B}: significant differences with GB; ^{C}: significant differences with GC;^{D}: significant differences with GD; GA: group A; GB: group B; GC: group C; GD: group D. One-way ANOVA with Sidak's post hoc test controlled for age and $p < 0.01$.

Supplementary Table 6. LC-HRMS data for statistically significant features identified in serum samples.

Metabolite	HMDB code	Formula	Monoisotopic Mass	Ion	*m/z*	Error (ppm)	Matched fragments (*m/z*)
Sebacic acid	HMDB0000792	$C_{10}H_{18}O_4$	202.1205	$[M-H]^-$	201.1118	0	111.0806; 139.1115; 183.1023; 201.1118
L-Tryptophan	HMDB0000929	$C_{11}H_{12}N_2O_2$	204.0898	$[M-H]^-$	203.0819	1	116.0508; 117.0535; 142.0666; 186.0555; 203.0819
D-Phenylalanine	HMDB0250791	$C_9H_{11}NO_2$	165.0789	$[M-H]^-$	164.0714	1	72.0088; 91.0554; 103.0547; 147.0463; 164.0714
Hydroxyphenyllactic acid	HMDB0000755	$C_9H_{10}O_4$	182.0579	$[M-H]^-$	181.0504	1	79.9941; 119.0493; 134.0371; 135.0446; 163.0396; 181.0504
Unknown	-	-	-	-	384.3468	-	-
Unknown	-	-	-	-	117.0562	-	-
Unknown	-	-	-	-	130.0872	-	-
Unknown	-	-	-	-	129.0560	-	-
Unknown	-	-	-	-	1398.7980	-	-
Unknown	-	-	-	-	1456.8410	-	-

Metabolites presented with their HMDB identification code, chemical formula, monoisotopic mass, identified ion, mass-to-charge ratio (*m/z*), and error. A tolerance of 10 ppm mass error was used in the matches. HMDB: Human Metabolome Database.

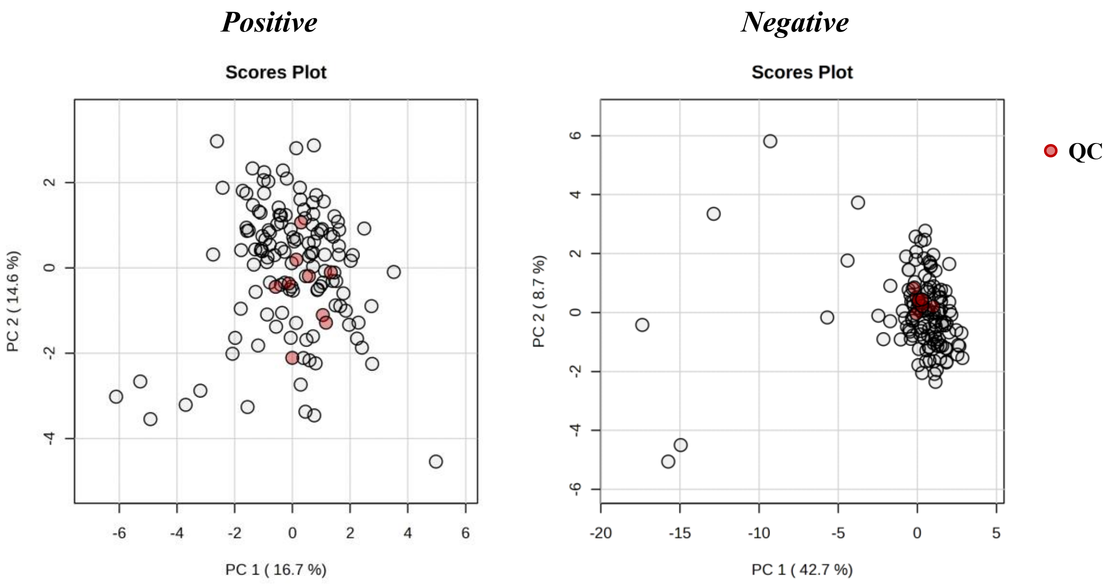

Supplementary Figure 1. Assessment of Instrumental stability using quality control (QC) samples. Data processed in *Metaboanalyst 6.0* software (https://www.metaboanalyst.ca/).

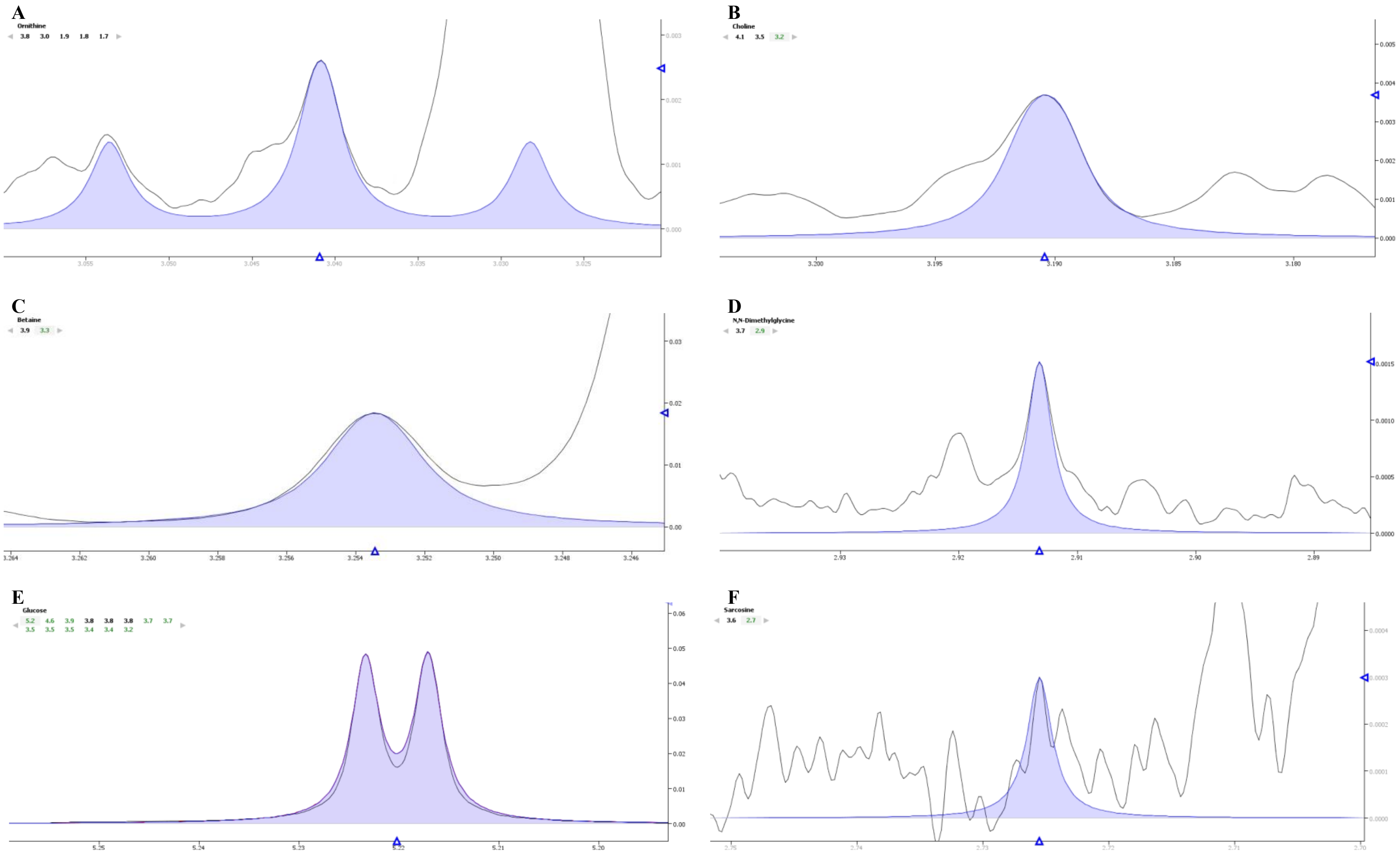

Supplementary Figure 2. Baseline adjustment performed with *Chenomx Suite 8.6* software and quantification of significant metabolites via ^{1}H NMR spectroscopy. A: Ornithine; B: choline; C: betaine; D: *N,N*-dimethylglycine; E: glucose; F: sarcosine
